# Variations in Glycogen Synthesis in Human Pluripotent Stem Cells with Altered Pluripotent States

**DOI:** 10.1371/journal.pone.0142554

**Published:** 2015-11-13

**Authors:** Richard J. Chen, Guofeng Zhang, Susan H. Garfield, Yi-Jun Shi, Kevin G. Chen, Pamela G. Robey, Richard D. Leapman

**Affiliations:** 1 Laboratory of Cellular Imaging and Macromolecular Biophysics, National Institute of Biomedical Imaging and Bioengineering, National Institutes of Health, Bethesda, MD, 20892, United States of America; 2 Experimental Carcinogenesis, National Cancer Institute, National Institutes of Health, Bethesda, MD 20892, United States of America; 3 NIH Stem Cell Unit, National Institute of Neurological Disorders and Stroke, National Institutes of Health, Bethesda, MD 20892, United States of America; 4 Craniofacial and Skeletal Diseases Branch, National Institute of Dental and Craniofacial Research, National Institutes of Health, Bethesda, MD, 20892, United States of America; University of Newcastle upon Tyne, UNITED KINGDOM

## Abstract

Human pluripotent stem cells (hPSCs) represent very promising resources for cell-based regenerative medicine. It is essential to determine the biological implications of some fundamental physiological processes (such as glycogen metabolism) in these stem cells. In this report, we employ electron, immunofluorescence microscopy, and biochemical methods to study glycogen synthesis in hPSCs. Our results indicate that there is a high level of glycogen synthesis (0.28 to 0.62 μg/μg proteins) in undifferentiated human embryonic stem cells (hESCs) compared with the glycogen levels (0 to 0.25 μg/μg proteins) reported in human cancer cell lines. Moreover, we found that glycogen synthesis was regulated by bone morphogenetic protein 4 (BMP-4) and the glycogen synthase kinase 3 (GSK-3) pathway. Our observation of glycogen bodies and sustained expression of the pluripotent factor Oct-4 mediated by the potent GSK-3 inhibitor CHIR-99021 reveals an altered pluripotent state in hPSC culture. We further confirmed glycogen variations under different naïve pluripotent cell growth conditions based on the addition of the GSK-3 inhibitor BIO. Our data suggest that primed hPSCs treated with naïve growth conditions acquire altered pluripotent states, similar to those naïve-like hPSCs, with increased glycogen synthesis. Furthermore, we found that suppression of phosphorylated glycogen synthase was an underlying mechanism responsible for altered glycogen synthesis. Thus, our novel findings regarding the dynamic changes in glycogen metabolism provide new markers to assess the energetic and various pluripotent states in hPSCs. The components of glycogen metabolic pathways offer new assays to delineate previously unrecognized properties of hPSCs under different growth conditions.

## Introduction

Human pluripotent stem cells (hPSCs) hold promise for cell-based therapies and cell replacements. Two types of hPSCs that include human embryonic stem cells (hESCs) and induced pluripotent cells (iPSCs) are currently undergoing extensive characterizations in order to understand their basic properties [1–6]. Unlike mouse embryonic stem cell (mESC) culture, which depends on growth medium supplemented with the leukemia inhibitory factor (LIF) and the bone morphogenetic protein (BMP-4) [7], hPSC culture relies on combinations of core signaling molecules such as FGF2, activin, nodal, and TGF β [8–11]. Thus, mESCs and hPSCs possess different pluripotent states, termed the naïve and primed state respectively [12]. Noticeably, the two different states can be interchangeable under specific growth conditions [13–18].

Current characterizations of hPSCs are based on genome-wide profiling and some biochemical analyses, which focus on microarray, gene copy number variations, microRNAs, and proteomics [4–6, 19, 20]. Although these assays and analyses contribute significantly to our current knowledge about hPSCs, they do not pertain to ultrastructural and biochemical changes related to cellular metabolism such as the utilization of glucose for energy production. Currently, there are only few studies on ATP generation, glycolytic states, and oxidative respiration in hPSCs [21, 22]. Interestingly, a glycolytic prevalence in hPSCs, similar to that of cancer cells, was found in primed hPSCs and epiblast-derived stem cells (EpiSCs), but not in naïve mESCs [21, 22]. These studies not only provide important insights into the role of glucose metabolism in controlling pluripotent states, but also encourage investigating other related metabolic pathways (such as glycogen synthesis) in maintaining the homeostasis and energetic balance of hPSCs.

Glycogen metabolism underlies many fundamental biochemical processes, which govern both physiological and pathological conditions, and which are involved in many important biochemical pathways relating to JAK/STAT signaling and the action of insulin [23]. Glycogen synthesis and storage are active in certain types of cells such as hepatocytes in liver and myocytes in skeletal muscle, but absent in some highly proliferative cancer cells [24]. One might speculate that hPSCs undergo very limited glycogen synthesis due to their high proliferation rates. Nevertheless, there is little knowledge about the glycogen utilization in either hESCs or iPSCs [25]. Lack of information on glycogen synthesis impedes our understanding of the essential energy states that maintain the pluripotency and self-renewal of hPSCs.

The assessment of glycogen metabolism often depends on indirect evidence such as enzymatic assays owing to the lack of specific antibodies for glycogen [26, 27]. Fortunately, glycogen aggregates display unique ultrastructures in transmission electron microscopy (TEM), thereby providing a powerful analytical tool for directly assessing glycogen synthesis. We have also developed techniques for preparing thin plastic-embedded TEM sample sections from cells attached to a plastic slide. These improvements allow for a quantitatively ultrastructural analysis of glycogen synthesis in cross sections of hPSCs. Using stereology principles, we were able to quantify the volume fractions of glycogen bodies within stem cells, and to delineate differences in glycogen distributions in hPSCs.

In this study, we combined TEM, immunofluorescence microscopy, and biochemical methods to study glycogen metabolism in hPSCs under different growth protocols [28, 29], including those recently developed naïve growth conditions [13]. Our findings help to define differential cellular and pluripotent states under various substrate, signaling, and culture conditions. In this way, we propose the glycogen state as a structural marker for delineating altered pluripotent states, unravelling previously unrecognized properties of hPSCs, and redefining energetic states during cellular differentiation.

## Materials and Methods

### Reagents, Antibodies, and Materials

The reagents used in this study include recombinant proteins: BMP-4 (Catalog number or Cat no: 314-BP-010) and FGF-2 (Cat no: 233-FB) purchased from R&D Systems (Minneapolis, MN) and recombinant human leukemia inhibitory factor (LIF) (Cat no: LIF1010) from Millipore (Temecula, CA). Antibodies include: anti-Oct-4 (mouse IgG2b; Cat no: sc-5279, Santa Cruz Biotechnology, CA); anti-phospho-glycogen synthase (pGS-ser641) antibody (Cat no: #3891), GSK-3β (3D10, mouse monoclonal antibody or mmAb, IgG2a, Cat no: #9832), and glyceraldehyde 3-phosphate dehydrogenase (GAPDH) rabbit mAb (14C10, Cat no: #2118) from Cell Signaling Technologies Inc. (Danvers, MA); rabbit anti-glycogen synthase (C-terminal) (Cat no: 04–357) and mouse anti-GAPDH (Cat no: MAB374) from Millipore. Secondary antibodies used for immunostaining comprise Alexa Fluor® 488 goat anti-mouse IgG2a (γ2a) (Cat no: A211312), Alexa Fluor®555 Goat anti-Rabbit IgG (Cat no: A21428), and Alexa Fluor® 647 Goat anti-Mouse IgG2b (Cat no: A21242) from Invitrogen Inc. (Calsbad, CA). Secondary antibodies used for Simple Western experiments were provided by ProteinSimple (San Jose, CA).

Chemical reagents: 2-(N-(7-nitrobenz-2-oxa-1,3-diazol-4-yl)amino)-2-deoxyglucose) or 2-NBDG, a fluorescent glucose derivative that has an excitation wavelength of 480 nm and emits on 535 nm, was obtained from Molecular Probes (Eugene, OR), dorsomorphin (BMP-4 inhibitor, abbreviated as BMP4i, Cat no: P5499), anhydrous dextrose (D-glucose), and Triton X-100 were purchased from Sigma-Aldrich (St. Louis, MO). BIO (inhibitor of the glycogen synthase kinase 3 or GSK3i, Cat no: BIO-025) was ordered from StemRD Inc. (Burlingame, CA); CHIR99021 (GSK3i, Cat no: 04–0004) and PD0325901 (inhibitor of the mitogen-activated protein kinase enzymes MEK1 and MEK2, abbreviated as MEKi, Cat no: 04–0006) from Stemgent (Cambridge, MA); and Y-27632 (inhibitor of the Rho-associated protein kinase p160ROCK or ROCKi; Cat no: 1254) from TOCRIS (Minneapolis, MN).

### Human Pluripotent Stem Cell Lines and Culture

There are 4 hESC and 2 iPSC lines used in this study. Human ES lines include H1 and H9 (NIH codes: WA01 and WA09) and their genetically modified reporter cell lines (H1 Oct4-EGFP and H9 hNanog-pGZ) (WiCell Research Institute, Madison, WI). The human iPSC lines BC1 and NIH-i12 were described previously [6, 30]. Both hESCs and iPSCs were plated on hESC-qualified Matrigel (BD BioSciences, Bedford, MA) and grown in mTeSR1 medium (StemCell Technologies, Vancouver, Canada). These cells were grown as monolayers to reduce the heterogeneity and also cultured as colonies on mouse embryonic fibroblast (MEF) feeder for comparison in the assays, essentially as previously described [28, 29]. For electron microscopic analysis, hPSCs were directly grown on Thermanox Plastic Coverslips coated with 2.5% Matrigel. For monolayer culture of hPSCs, 10 μM ROCKi (Y-27632) was used for enhancing initial single-cell plating efficiency [28, 29]. In our pilot experiments, the hPSCs were initially treated with 100 ng/mL of BMP-4 and 3 μM GSK3i (CHIR99021) for 48 hours to examine whether inhibition of the two major signaling pathways (related to BMP-4 and glycogen synthesis) could lead to cellular differentiation or alterations in pluripotent states. These treated cells were directly fixed with proper chemical solutions for structural analysis under TEM and immunofluorescence microscope.

### Cell Growth Conditions toward Naïve Pluripotent States

To facilitate a comparative analysis of glycogen variations between different pluripotent states, we cultivated hPSCs under naïve cell growth conditions using a well-established protocol [13]. This method is similar to our current hPSC growth platform that utilizes Matrigel and mTeSR1 medium, except the addition of the naïve component 3iL in the medium. The 3iL includes 2 μM GSK3i (BIO), 1 μM MEKi (PD032591), 2 μM BMP4i (dorsomorphin), and 10 ng/mL of LIF [13]. This naïve growth protocol enables a rapid conversion (2–4 days) of primed hPSCs (grown in mTeSR1 medium on Matrigel) to a naïve or naïve-like pluripotent state. To unravel the key components that regulate glycogen synthesis, we also compared glycogen variations in hPSCs under different combinations of the above naïve components, i.e. 2 μM GSK3i (BIO), 2i (2 μM GSK3i + 1 μM MEKi), 3i (2i + 2 μM BMP4i), 2iL (2i + 10 ng/mL LIF), and 3iL (3i + 10 ng/mL LIF) for 48 hours prior to assays.

### Glycogen Colorimetric Assay

**Fig 1:**
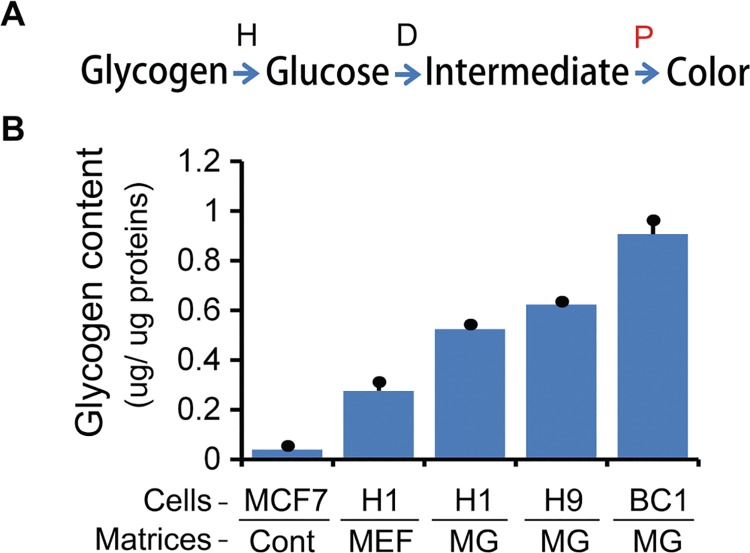
Variations in glycogen synthesis in human pluripotent stem cells (hPSCs). (A) Schema of the glycogen assay based on the protocol from BioVision Inc.: glycogen was hydrolyzed (H) into glucose, then glucose developed (D) into an intermediate and reduce probe (P) to produce color products. (B) Determination of glycogen content in hPSCs grown under various growth conditions. Column 1: MCF7 breast cancer cells used control (Cont) for the assessment of glycogen levels; Column 2: H1 colonies grown on MEF as previously descried in Materials and Methods. The glycogen content was measured at cell passage number 38. Column 3: H1 colonies initially grown on MEF for 35 passages followed by growing on 2.5% BD Matrigel (MG) in mTeSR1 for 5 passages. Column 4: H9 cells were initially grown on MEF for 32 passages followed by passaging on 2.5% BD Matrigel (MG) in mTeSR1 medium for 9 passages. Column 5: BC1 human iPSCs were initially grown on MEF for 50 passages followed by passaging on 2.5% BD Matrigel (MG) in mTeSR1 medium for 26 passages.

Glycogen content was determined by using a glycogen colorimetric method (Colorimetric Assay Kit II, Cat no: K648-100, BioVision Inc., Milpitas, CA) following manufacturer’s instruction (Fig 1A). Briefly, cell pellets were homogenized with 100 μl of ice-cold glycogen hydrolysis buffer on ice for 10 minutes. The supernatants were collected after micro-centrifugation at 12,000 *rpm* for 5 minutes. Protein concentration was determined by the bicinchoninic acid (BCA) protein assay (Prod number: 23225, Thermo Scientific, Rockford, IL). Glycogen in hPSC lysates, MCF7 breast cancer cell lysates (control), and glycogen standards was hydrolyzed into glucose at room temperature for 30 minutes. The glucose was then oxidized to form an intermediate that reduces a colorless probe to a colored product also at room temperature for another 30 minutes. Samples without a glycogen hydrolysis step were used as controls to assess the contribution of background glucose to reduced final products. The absorbance of glycogen products was measured at 450 nm with a SpectraMax Plus384 microplate reader (Molecular Device, Sunnyvale, CA). The glycogen content was finally calculated by subtracting glucose background from all samples and normalized to protein input.

### Transmission Electron Microscopy (TEM)

The hESC and iPSC cells were grown on Thermanox@ Plastic Coverslips (Cat no: 174950, Lot no: 1059846; Thermo Fisher Scientific, Rochester, NY) for electron microscopic analysis. These cells were fixed in a mixture of 2.5% paraformaldehyde and 2.0% glutaraldehyde in PBS for 1 hour to stabilize ultrastructure, followed by extensive washing in PBS. Samples were then rinsed in 0.1 M sodium cacodylate buffer (SCB; pH = 7.4) and post fixed in 1.0% osmium tetroxide plus 0.8% potassium ferricyanide in the above buffer for 60 minutes. After several additional rinses in SCB, the samples were dehydrated in a series of ethanol concentrations (30%, 50%, 75%, 95% for 5 min and 100%) in water for 20 minutes, each with three changes. The samples were subsequently infiltrated with Epon-Aradite (Ted Pella, Redding, CA) for one day (50% Epon-Aradite and 50% ethanol for 1 hour, 75% Epon-Aradite and 25% ethanol for 1 hour, and 100% Epon-Aradite for overnight). Samples were then polymerized at 60°C for one day.

Ultrathin sections (about 80 nm in thickness) were cut on a Leica EM UC6 Ultramicrotome (Leica, Buffalo Grove, IL) and collected on copper slot grids. Sections were counterstained with uranyl acetate and lead citrate, and examined with an FEI Tecnai T12 transmission electron microscope operated at 120-keV beam energy. Images were acquired digitally using a Gatan 2k x 2k pixel cooled CCD camera.

### Stereological Sampling and Planimetry-based TEM Analysis

Specimens were analyzed in an FEI Tecnai^TM^ T12 transmission electron microscope (TEM). Images were captured using an Ultrascan 2k x 2k CCD camera and Gatan Digital Micrograph software. Data were collected using stereology principles to sample the stem cells as randomly as possible, and no assumptions were made about the orientation and distribution of the cells in the cross sections. Image contrast and magnifications were adjusted to visualize the ultrastructure of the glycogen bodies.

Planimetry analysis was performed using the ImageJ program (National Institutes of Health, Bethesda, MD) to draw contours around the regions of interest (ROI), which could be used to calculate the areas in square micrometers. Glycogen content in each cell was determined by computing the ratio between the cumulative areas of glycogen bodies and the total area of the cell section. A total of 50 cells were analyzed to provide sufficient statistical power in the analysis.

### Immunofluorescence Microscopy

Human hPSCs were fixed in 4% paraformaldehyde solution at room temperature for 20 minutes and then permeabilized with 0.1% Triton X-100 in D-PBS solution. The background fluorescence in the cells was blocked with 10% normal goat serum (Sigma-Aldrich). The samples were initially incubated with desired primary antibodies for 2 hours, rinsed three times in D-PBS; and further incubated with secondary antibodies for 1 hour. After three washes in D-PBS, the cells were stained with the Hoechst 33342 solution, mounted, and examined under a Zeiss Axiovert microscope (Zeiss, Germany). The fluorescence images were analyzed by the ImageJ program. The mean fluorescence intensity of each cell was measured in 100 to 200 individual cells randomly assigned from phase images.

### 2-NBDG Accumulation and Retention Assays

The fluorescent glucose derivative 2-NBDG has been widely used for monitoring glucose uptake and retention [31, 32]. Due to the integrative capacity of 2-NBDG into glycogen polymer in cell culture, it is particularly useful for imaging glycogen synthesis [31]. To assay 2-NBDG accumulation, hPSCs were simply incubated with 100 μM 2-NBDG in the presence of 10 mM D-glucose for 2 hours. The cells were refreshed with drug-free medium and quickly imaged under fluorescence microscope using unsaturated time exposure. To monitor 2-NBDG glycogen labeling, 2-NBDG was removed from the culture medium. The cells were further incubated with 10 mM D-glucose for another 2 hours, washed in D-PBS, and finally fixed in 4% paraformaldehyde prior to imaging analysis. In this study, we used the H1 Oct4-EGFP reporter cell line and a well-characterized non-EGFP iPSC line (i.e., NIH-i12) in the 2-NBDG assay.

The use of H1 Oct4-EGFP reporter line with 2-NBDG would enable us to perform real-time imaging of 2-NBDG accumulation and retention at desired time points and evaluate altered pluripotent states simultaneously. In this way, we are able to avoid the use of cumbersome and less applicable immunofluorescence staining of fixed samples. The 2-NBDG fluorescence signal could be readily differentiated from Oct4-EGFP, because (i) 2-NBDG has much stronger fluorescence intensity than Oct4-EGFP and (ii) the punctate localization or foci-like retention patterns of 2-NBDG are different from that uniform EGFP signal. In addition, the use of Oct4-EGFP alone as a basal fluorescence control in this study has greatly facilitated the fluorescence signal discrimination and statistical subtraction.

### Simple Western Experiments

We used a Simple Western system (Simon™, ProteinSimple, San Jose, CA), a non-gel based and Western blot-like substitute, to efficiently and quantitatively analyze protein expression. This reinvented and partially automated machine performs size-based separation after sample loading, immunoprobing, washing, detection, and quantitative data analysis. Briefly, cell pellets were resuspended in RIPA buffer (Cat no: R0278, Sigma) in the presence of a protease and phosphatase inhibitor cocktail (Cat no: 1861281, Thermo Scientific). The supernatants were collected, aliquoted, and stored in -80°C prior to use. Freshly made 1 M DTT was used to reduce all samples during denaturation. The biotinylated ladder was used for molecular weight determination and run in capillary 1 (lane 1), which contains 6 proteins at molecular weights of 12, 40, 66, 90, 116 and 180 kDa. Protein lysates (~5 μg per lane) were loaded into each capillary and run together with fluorescent standards, which permit molecular weight normalization to the biotinylated ladder. Proteins of interest (such as glycogen synthase and glycogen synthase kinase) were identified using specific primary antibodies, probed with HRP-conjugated secondary antibodies, detected or quantitated by chemiluminescent signals. Protein expression was normalized to GAPDH levels.

### Statistical Analyses

Statistical analyses were performed with Microsoft Excel 2000 and with GraphPad Prism 6 (GraphPad Software, Inc., La Jolla, CA). All Box-and-Whisker plots were created by GraphPad Prism 6. A two-tailed and unpaired Student *t*-test was used in this study.

## Results

### Variations of Glycogen Synthesis in hPSCs under different Growth Platforms

**Fig 2:**
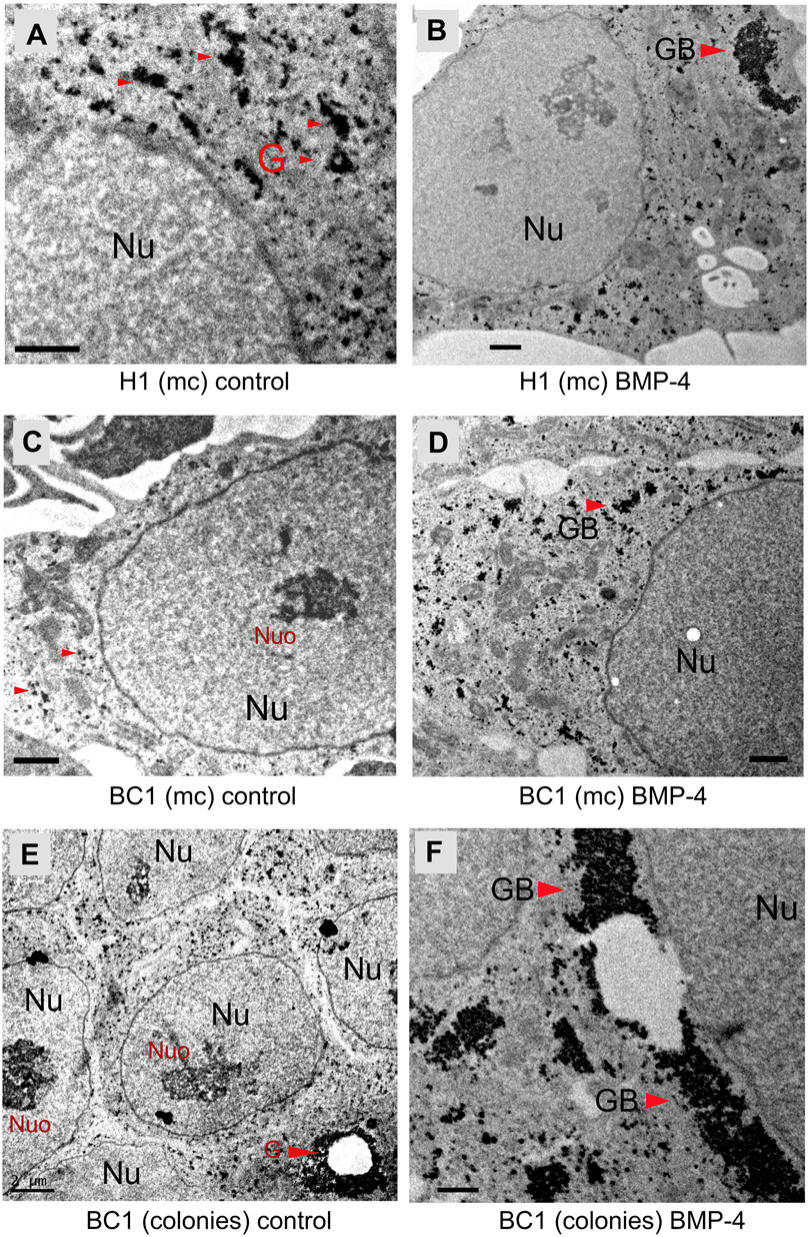
Transmission electron micrograph (TEM) analysis of glycogen synthesis in hPSCs. (**A, C**) Representative images of untreated H1 and BC1 cells grown as a non-colony type monolayer (mc) on 2.5% Matrigel. (**E**) Untreated BC1 cells grown as colonies on Matrigel. (B, D, F) Representative images of hPSCs (left panel) treated with 100 ng/mL of BMP-4. The red colored arrowheads indicate stained glycogen aggregates in the cytoplasm of the cell. Abbreviations: G, glycogen particles or aggregates; GB, glycogen bodies; Nu, the nucleus of the cell; Nuo, the nucleolus of in the nucleus. Scale bars represent 1 μm in A, B, C, D, and F and 2 μm in E.

We compared glycogen content in multiple hPSC lines grown under different growth conditions. MCF7 breast cancer cells, having a consistent glycogen content (~40 ng/μg protein) by the glycogen colorimetric assay [33], were used as a control for the assessment of glycogen levels. In general, hPSCs grown as colonies had at least 7-fold higher glycogen content than MCF7 control (Fig 1B, *P* < 0.05). Both H1 and H9 colonies grown on 2.5% Matrigel showed approximately 2-folder higher glycogen content than H1 colonies on MEF feeder (Fig 1B, *P* < 0.05). Moreover, BC1 iPSCs on Matrigel showed 3.3-fold higher glycogen content than H1 colonies on MEF (Fig 1B: columns 2 and 5, *P* = 0.0002). Under TEM images, we confirmed that there are glycogen aggregates in both hESCs (e.g., H1 cells, Fig 2A, and S1 Fig) and iPSCs (e.g., BC1 cells, Fig 2C, 2E, and S2 Fig), which were sensitive to induction by the differentiation factor BMP-4 (Fig 2B, 2D and 2F). We also observed that the growth patterns of BC1 iPSCs have a significant impact on glycogen synthesis when these cells are treated with BMP-4. It appeared that BC1 cells grown as colonies on plastic coverslip had a greater glycogen accumulation than the cells cultured as non-colony type monolayer (Fig 2D and 2F). These data demonstrate the greater variability of glycogen synthesis under different growth platforms. Different growth patterns affect BMP-4-mediated glycogen synthesis, suggesting that other signaling pathways may also impact on the energetic states.

### Pharmacological Inhibition of GSK-3 Induces the Formation of Glycogen Bodies

**Fig 3:**
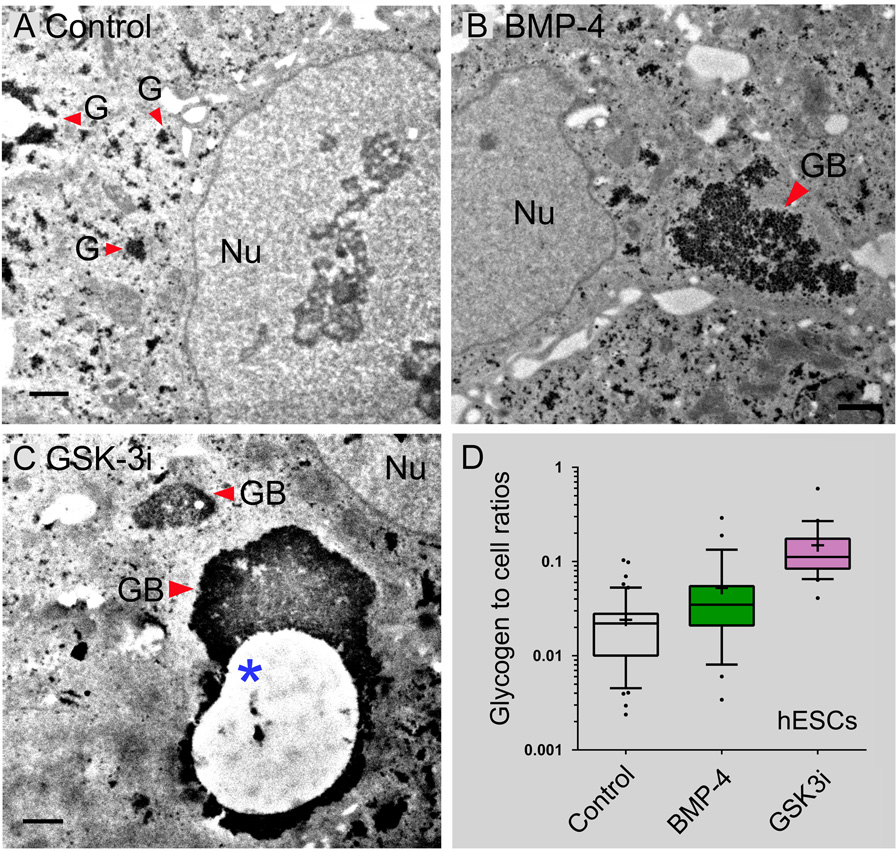
TEM analysis of glycogen synthesis and the formation of glycogen bodies mediated by BMP-4 and GSK-3 inhibition in hESCs. (**A**) Glycogen body formation in untreated H1 cells (control). (**B**) Glycogen body formation in BMP-4 treated H1 cells. (**C**) Glycogen body formation in GSK3i (CHIR99021)-treated H1cells. The asterisk sign indicates a glycogen defect region in the glycogen body, which likely resulted from dissociation of glycogen aggregates when the specimens were floating in solution during sample preparation. Additional TEM micrographs are available in Supporting Information S1 Fig and S2 Fig. (**D**) Box-and-Whisker plots of glycogen to cell ratios (with 10–90% percentile) in hESC control (n = 49 cells), BMP-4 treated cells (n = 29), and GSK3i treated cells (n = 22). The statistics included both H1 and H9 cells to increase statistical power. All hESC (i.e., H1 and H9) cells were grown as a non-colony type monolayer (mc) on 2.5% BD Matrigel in meTeSR1 medium and then treated with 100 ng/mL of BMP-4 and 3 μM GSK3i for 48 hours. The red-colored arrowheads indicate the formation of glycogen bodies (GB) in the cytoplasm of the cell. The plus signs (+) in the plots indicate the location of mean values. Abbreviations: GB, glycogen bodies with defined boundaries; Nu, the nucleus of the cells. Scale bars in (A, B) represent 2 μm; and scale bars in (C) represent 1 μm.

GSK3 phosphorylates glycogen synthase, thereby inhibiting its activity, and resulting in decreased glycogen accumulation. In contrast, GSK3 inhibition would result in dephosphorylation of glycogen synthase and thus an increased glycogen accumulation. We further reasoned that the enzymatic activity that directly regulates glycogen synthase might have greater influences on glycogen consumption and storage. To test this hypothesis, we treated H1 cells (grown as monolayer) with 3 μM CHIR99021 (i.e., GSK3i), a potent inhibitor of glycogen synthase kinase 3 (GSK-3). Both untreated H1 and BMP-4-treated H1 cells were used as controls for comparison in this experiment (Fig 3A and 3B). Massive glycogen bodies were frequently found in the cytoplasm of GSK3i-treated cells (Fig 3C). Our results indicated that glycogen synthesis and the formation of glycogen bodies were significantly increased by 10.7% in hESCs cells treated with GSK3i (mean ± s.d. = 15.9 ± 11.2%) compared with those BMP-4-induced cells (mean ± s.d. = 5.2 ± 6.2%) (*P* = 0.0012) (Fig 3D). These data suggest that the altered glycogen metabolism in hPSCs may be associated with the inhibition of enzymatic activity of GSK-3, which then directly regulates the activity of glycogen synthase.

### Modulation of Glycogen Storage might be Independent of Differentiation

**Fig 4:**
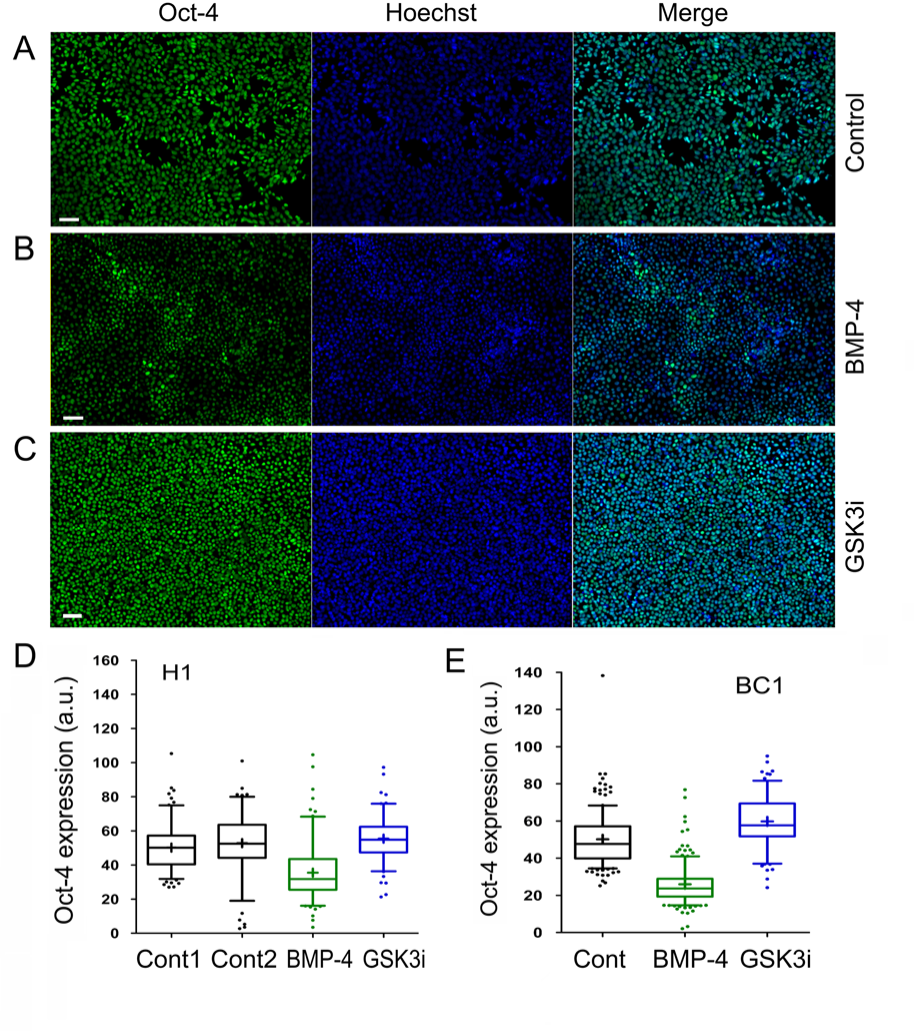
Immunofluorescence analysis of the expression of the pluripotent marker Oct-4 in human pluripotent stem cells (hPSCs). Oct-4 expression in H1 control cells (A), H1 cells treated with 100 ng/mL of BMP-4 (B), and H1 cells treated with 3 μM GSK3i (CHIR99021) for 48 hours (C). The cellular genomic DNAs were stained by the Hoechst 33342 dye (Hoechst). The images were collected with a fluorescence microscope (Zeiss). (**D** and **E**) Box-and-Whisker plots of Oct-4 expression (with 5–95% percentile) in both H1 and BC1 cells under the indicated treatments. Control 2 (Cont2) in D is an additionally untreated control of H1 cells. The plus signs (+) in the plots indicate the location of the mean values determined from 116 to 150 individual cells by the ImageJ program. One of two independent experiments is shown. Scale bars represent 50 μm.

To further verify the relationship between glycogen utilization and the pluripotent state of hPSCs, we monitored the expression of the pluripotent factor Oct-4 (Fig 4). Our data indicate that the average Oct-4 immunofluorescence intensity in H1 control cells (i.e., mean ± s.e.m. = 50.2 ± 1.0; n = 150 cells) was significantly reduced in BMP-4-treated cells (i.e., mean ± s.e.m. = 35.5 ± 1.3; n = 150 cells) (Fig 4D: columns 1 and 3; unpaired *t*-test, two-tailed, *P* < 0.0001). Similar Oct-4 expression patterns were also found in BC1 cells treated with BMP-4 (Fig 4D: columns 1 and 2). These data suggest that BMP-4 inhibits Oct-4 expression, consistent with BMP-4 functioning as a differentiation factor under stem cell culture conditions [34].

In contrast, GSK3i-mediated glycogen synthesis was apparently associated with an elevated Oct-4 level. The average Oct-4 immunofluorescence intensity in H1 control cells was significantly upregulated in GSK3i-treated cells (i.e., mean ± s.e.m. = 55.5 ± 1.2; n = 123 cells; s.e.m., stands for standard error of the mean) (Fig 4D: columns 1 and 4; unpaired *t*-test, two-tailed, *P* = 0.0008). More pronounced Oct-4 upregulation was also confirmed in BC1 cells (Fig 4E: columns 1 and 3). Taken together, our data suggest that the increase in glycogen synthesis or the formation of glycogen bodies seems to be independent of cellular differentiation of hPSCs.

### Elevated Glycogen Synthesis during the Transition to Naïve Pluripotent States

**Fig 5:**
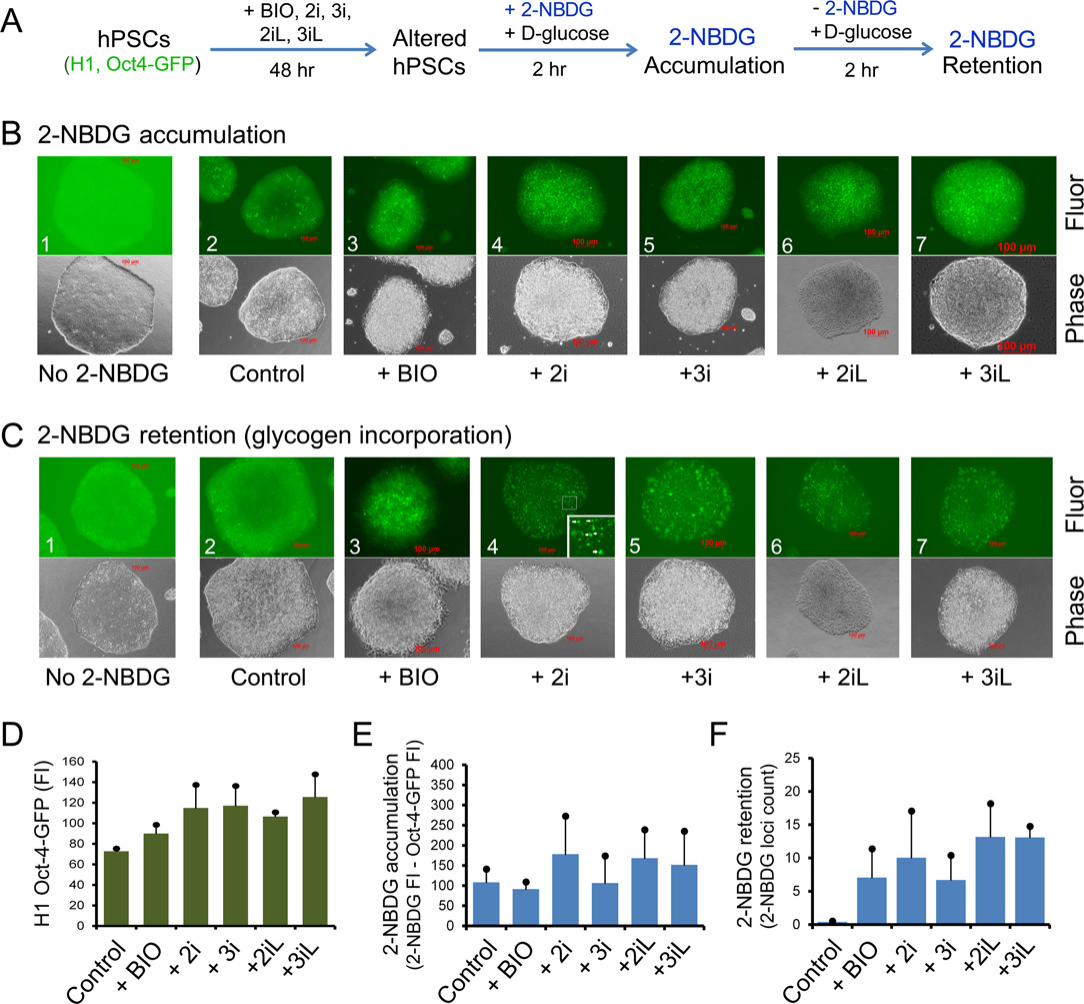
2-NBDG accumulation and retention in H1 Oct4-EGFP under naïve hPSC growth conditions. (**A**) Schema of 2-NBDG accumulation and retention (glycogen labeling) experiments. (**B**) 2-hour 2-NBDG accumulation in the presence of 10 mM D-glucose. Upper panel: green fluorescence intensity (Fluor) images that include signals from both Oct4-EGFP and 2-NBDG. These images were obtained (immediately after replacing with fresh mTeSR1 medium) by non-saturated time-exposure guided by an autoexposure software (Zeiss Inc.). No 2-NBDG indicates fluorescence background images produced from Oct4-EGFP signal without the use of 2-NBDG. The fluorescence intensity was determined by the signal/noise ratio between cellular fluorescence (signal) and background (noise). The fluorescent background in the 2-NBDG control was due to non-saturated auto exposure using the Zeiss Axiovert imaging system. Lower panel: the corresponding phase images of the upper panel. Only brightness was adjusted in phase images (presented in both B and C) to enhance the image presentation in this figure. (**C**) 2-NBDG retention and glycogen labeling carried out in the presence of 10 mM D-glucose and absence of 2-NBDG. Upper panel: unique fluorescence loci (dots) were derived from 2-NBDG signals as indicated by arrows in the inset of Fig 5C4. Lower panel: the corresponding phase images of the upper panel. (**D**) Quantitative analysis of Oct4-EGFP signals without the addition of 2-NBDG. **(E**) Quantitative analysis of 2-NBDG accumulation in Fig 5B. (**F**) Quantitative analysis of 2-NBDG retention and glycogen labeling by counting 2-NBDG loci as presented in Fig 5C. Columns represent mean fluorescence intensity measured from at least 4 random colonies and bar standard deviations. Abbreviations (depicted sequentially): BIO, 2 μM GSK3i (BIO); 2i, 2 μM BIO + 1 μM MEKi; 3i, 2i + 1 μM BMP4i; 2iL, 2i + 10 ng/mL LIF; 3iL, 3i + 10 ng/mL LIF; 2-NBDG, (2-(N-(7-nitrobenz-2-oxa-1,3-diazol-4-yl)amino)-2-deoxyglucose), a fluorescent glucose derivative, overlapping with EGFP signals. Scale bars represent 100 μm.

As suggested from the above results, GSK-3 inhibition by GSK3i (CHIR99021) in ROCKi-mediated monolayer culture resulted in increase in glycogen synthesis and elevated Oct-4 expression, suggesting these hPSCs acquired an altered pluripotent state. To verify whether there is also altered glycogen synthesis presented in hPSCs with different pluripotent states, we modified primed hPSCs under our current culture protocols by treating them with the naïve hPSC culture components (3iL) included in a well-established protocol (Fig 5A) [13]. We systematically analyzed glycogen variations in hPSCs under BIO, 2i, 3i, 2iL, and 3iL. As indicated in the phase images of Fig 5B, there were progressive morphological changes after 48-hour treatments of H1 Oct4-EGFP cells with above conditions. These treated cells were morphologically similar to those described naïve cells [13]. Moreover, these cells did not commit to lineage differentiation as monitored by increased Oct4-EGFP expression in these cells (Fig 5B and 5D). There are 1.2-, 1.6-, 1.6-, 1.5-, and 1.7-fold elevations in Oct4-EGFP expression compared to non-treated control, respectively under BIO, 2i, 3i, 2iL, and 3iL culture conditions (Fig 5D). Even a 1.2-fold increase in Oct-EGFP expression in BIO-treated cells (compared to non-treated control) is considered to be significant (Fig 5D: columns 1 and 2, *P* = 0.008). Thus, these data suggest that H1 Oct4-EGFP cells treated with naïve growth conditions acquire altered pluripotent states, similar to those previously described naïve-like hPSCs [13].

To monitor glycogen synthesis in the above treated cells, we labeled the cells with 2-NBDG, a fluorescent D-glucose derivative that can be incorporated into glycogen chains in live cells [31, 32]. After 2-hour accumulation of 2-NBDG in the presence of 10 mM D-glucose, we observed that 2i, 2iL, and 3iL had 1.6-, 1.6, and 1.4-fold enhanced accumulations of 2-NBDG respectively, compared to control, but with *t*-test *P* values greater than 0.05 (Fig 5E). Thus, the significance of enhanced uptake of 2-NBDG under these naïve growth conditions remains to be determined, preferentially in a larger dataset. In the case of glycogen labeling experiments, after the removal of 2-NBDG, all treated cells showed 17- to 33-fold increase in 2-NBDG loci (Fig 5F: columns 2 to 6; *P* < 0.05). Noticeably, the addition of LIF to 3i enhanced 2-NBDG retention by 2-fold (Fig 5F: lanes 4 and 6; *P* = 0.03). These data suggested that elevated 2-NBDG levels in these naïve-like cells are due to increased 2-NBDG incorporation during glycogen synthesis in the cells. The presence of LIF might stabilize glycogen synthesis.

**Fig 6:**
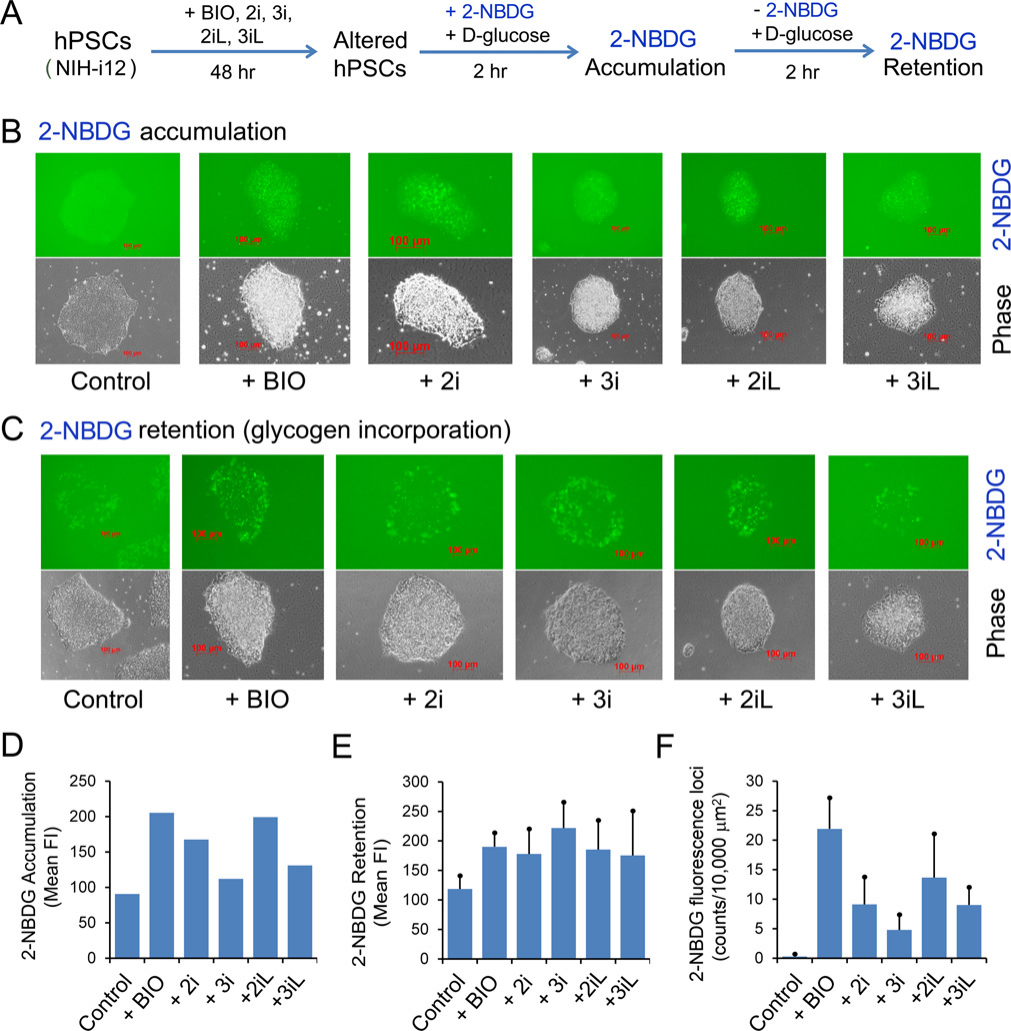
2-NBDG accumulation and retention in NIH-i12 iPSCs under naïve hPSC growth conditions. (**A**) Schema of 2-NBDG accumulation and retention (glycogen labeling) experiments. (**B**) 2-hour 2-NBDG accumulation in the presence of 10 mM D-glucose. Upper panel: green fluorescence intensity (Fluor) images from 2-NBDG alone. These images were obtained (immediately after replacing with fresh mTeSR1 medium) by non-saturated time-exposure guided by an autoexposure software (Zeiss Inc.). Lower panel: the corresponding phase images of the upper panel. Only brightness was adjusted in phase images (presented in both B and C) to enhance the image presentation in this figure. (**C**) 2-NBDG retention and glycogen labeling carried out in the presence of 10 mM D-glucose and absence of 2-NBDG. Upper panel: unique fluorescence loci (dots) were derived from 2-NBDG signals as detailed in Fig 5. (**D**) Quantitative analysis of mean fluorescence intensity (FI) in Fig 6B. (**E**, **F**) Quantitative analysis of 2-NBDG retention and glycogen labeling by measuring mean fluorescence intensity (FI, arbitrary units) from at least 4 random colonies (E) and by counting 2-NBDG loci (F). Columns represent mean fluorescence intensity measured from at least 4 random colonies and bar standard deviations. Scale bars represent 100 μm.

To reproduce the effects of naïve growth conditions on 2-NBDG retention in iPSCs, we performed the same experiment in NIH-i12, a well-characterized iPSC line reported in the NIH StemCellDB [6]. As revealed in Fig 6D, NIH-i12 cells showed similar accumulation and retention patterns to those of H1 Oct4-EGFP cells presented in Fig 5. One interesting difference between the two cell lines is that BIO had a pronounced effect on 2-NBDG accumulation (Fig 6D: columns 1 and 2), with 1.5 to 1.9-fold elevation in 2-NBDG fluorescence retention (Fig 6E: columns 2 to 5, *P* < 0.05), and 78-fold increase in 2-NBDG retention based on 2-NBDG fluorescence loci counts (Fig 6F: columns 1 and 2, *P* = 0.004).

**Fig 7:**
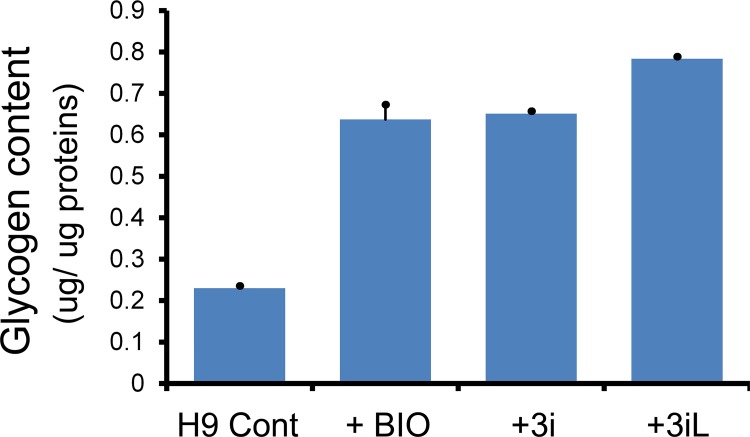
Glycogen colorimetric assays of H9 NANOG reporter (H9 hNanog-pGZ) cells under naïve hPSC growth conditions. The reporter cells (abbreviated as H9 Cont) were treated with GSK3i (BIO), 3i (GSK3i + MEKi + BMP4i), and 3iL (3i + LIF) as described in Materials and Methods. Glycogen colorimetric assays were performed under the same condition as described in Fig 1.

In addition, using the glycogen colorimetric assay, we were able to show that hPSCs (e.g., H9 hNanog-pGZ cells) treated with some naïve growth conditions (e.g., BIO, 3i, and 3iL) had 2.8- to 3.4-fold increase in glycogen content compared with control (Fig 7, *P* < 0.05), a pattern similar to the 2-NBDG retention patterns we observed in H1 Oct4-EGFP and NIH-i12 cells (Figs 5 and 6). Taken together, these data revealed that some naïve growth conditions may have a potential role in modulating glycogen metabolisms.

### Altered Expression and Function of Glycogen Synthase underlying Glycogen Changes in hPSCs with Different Pluripotent States

**Fig 8:**
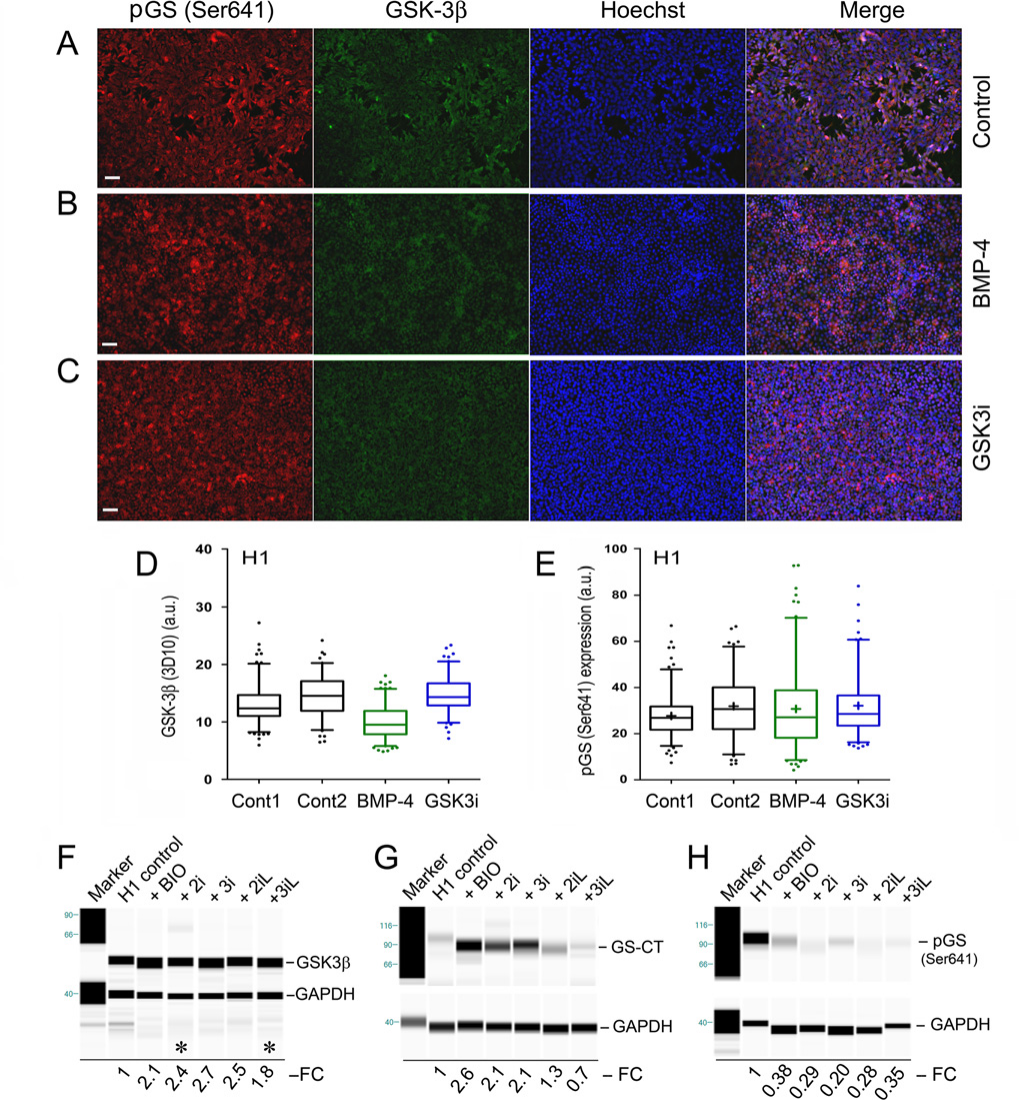
Immunofluorescence analysis of coexpression of the phospho-glycogen synthase (pGS-Ser641) and GSK-3β in hPSCs. Coexpression of pGS-Ser641 and GSK-3β in H1 control cells (**A**), H1 cells treated with 100 ng/mL of BMP-4 (**B**), and 3 μM GSK3i (CHIR99021) (**C**) for 48 hours. The cellular genomic DNAs were stained by the Hoechst 33342 dye (Hoechst). The images were collected with a fluorescence microscope (Zeiss). (**D** and **E**) Box-and-Whisker plots (with 5–95% percentile) of H1 cells under the indicated treatments: Control 2 (i.e., Cont 2) in both D and E is an additionally untreated control of H1 cells. The plus signs (+) in the plots indicate the location of mean values determined from 116 to 150 individual cells by the ImageJ program. One of two independent experiments is shown. Scale bars in the images represent 50 μm. (**F-H**) Simple Western experiments: H1 Oct4-EGFP (abbreviated as H1) cells were cultured under the monolayer culture condition for 2 passages and treated with naïve growth conditions as indicated for 48 hours. Simple Western was carried out in H1 Oct4-EGFP cell lysates using specific antibodies against GSK-3β, glycogen synthase (C-terminal), and phospho-glycogen synthase at Ser641 (pGS-Ser641). Protein expression was normalized to GAPDH protein control run in the same capillary (lane). Fold changes (FC) of protein expression related to untreated controls were labeled beneath each lane. The lanes marked by asterisk signs (in Fig 8F) were the results obtained from a separate experiment.

To verify whether GSK-3 activity was actually affected by both BMP-4 and GSK3i, we performed immunostaining of the same set of cells with specific antibodies that recognize GSK-3β and phospho-glycogen synthase (pGS-Ser641) (Fig 8A–8C). Our data indicate that the average GSK-3β immunofluorescence intensity in H1 control cells (i.e., mean ± s.e.m. = 12.2 + 0.27; n = 150 cells) was significantly reduced in BMP-4-treated cells (i.e., mean ± s.e.m. = 10.0 ± 0.23; n = 150 cells) (Fig 8D: columns 1 and 3; unpaired *t*-test, two-tailed, *P* < 0.0001). These data suggest that BMP-4-induced glycogen body formation acts through the inhibition of GSK-3.

However, GSK-3β down-regulation was not associated with down-regulation of the phospho-glycogen synthase in these cells (Fig 8E), suggesting that BMP-4 mediated glycogen synthesis was only partially associated with the GSK-3β pathway. Interestingly, GSK3i-mediated glycogen body formation was not directly associated with the GSK-3β-glycogen synthase pathway because the levels of both proteins were not apparently affected under the potent pharmacological GSK3i CHIR99021 (Fig 8E: columns 1, 2, 4). Additional mechanisms that involve different GSK-3β isoforms and/or various phosphorylation sites of glycogen synthase might underlie the molecular mechanisms of CHIR99021-mediated glycogen synthesis.

We employed Simple Western technologies to systematically analyze enzymatic changes related to glycogen synthesis in H1 Oct4-EGFP cells. GSK-3β is a major target of GSK-3 inhibition. Usually, GSK-3 inhibitors exert their inhibitory roles on GSK-3 by suppressing its function. As shown in Fig 8F, BIO-mediated GSK3 inhibition is not via down-regulation of GSK-3β. However, it enhanced GSK-3β protein expression by 2.1-fold (Fig 8F). All naïve growth conditions showed similar patterns of GSK-3β expression (Fig 8F). Furthermore, glycogen synthase (GS) is the direct downstream effector of GSK3 inhibition. As revealed in Fig 8G, GS was indeed affected and elevated in BIO-, 2i-, and 3i-treated cells, but not in the 3iL condition, which may be partially associated with increased GS in these conditions. Interestingly, BIO and its associated naïve growth conditions greatly reduced an inactive form of GS (i.e., pGS-Ser641) by 62–80% (Fig 8H). Of note, similar protein expression pattern was not revealed in CHIR99021-treated H1 cells by conventional immunofluorescence staining (Fig 8A and 8C). Nevertheless, our data suggest that altered glycogen synthesis in BIO and its associated naïve growth conditions may be due to decreased expression of inactive forms of GS, particularly the pGS at Ser641.

## Discussion

With respect to cellular metabolism of hPSCs, there is very limited information on this important topic. Although hPSCs have functional respiratory complexes that are able to implement oxidative respiration [35], ATP generation in hPSCs was largely produced by glycolysis [22, 35]. Further, there are pronounced metabolic differences between distinct pluripotent states. Primed ES cells (such as EpiSCs and hESCs) showed a highly glycolytic phenotype, whereas naïve mESCs utilize bivalent energy production [22, 35]. This glycolytic prevalence in hPSCs is reminiscent of the Warburg effect, in which it is observed that almost all cancer cells prefer high rates of glycolysis for their proliferation even under aerobic conditions [36, 37]. At least two molecular pathways that involve uncoupling protein 2 (UCP2) and hypoxia-inducible factor 1α (HIF1α) were shown to regulate the glycolysis-mitochondrial respiration transition. Both UCP2 and HIF1α avert mitochondrial glucose oxidation, thus facilitating the glycolytic pathway in undifferentiated hPSCs [22, 35].

Despite the insights into the glycolytic pathways in hPSCs, glycogen synthesis, another major aspect of glucose metabolisms, has not been previously studied in hPSCs under different pluripotent states. In the present study, we have revealed several new features of hPSCs in terms of their glycogen metabolism. These findings include differential glycogen accumulation under various growth differentiation conditions. The variations of glycogen synthesis in hPSCs also suggest metabolic differences between distinct pluripotent states in hPSCs.

### Differentiation-dependent Glycogen Synthesis Mediated by BMP-4 in hPSCs

When hPSCs are grown in mTeSR1 medium based on FGF2 and TGFβ signaling [28, 29], they possess predominantly the primed pluripotent state. Under this condition, BMP-4 is a potent differentiation factor for hPSCs [28, 38]. Thus, increased glycogen synthesis under this condition was coordinately regulated by differentiation signaling molecules such as BMP-4. These data suggest that glycogen synthesis may be a sensitive surrogate for lineage-related differentiation under this specific condition. Moreover, different growth patterns or modes affect BMP-4-mediated glycogen synthesis, further suggesting that hPSC culture methods may impact on the energetic states that are associated with glycogen storage.

### Differentiation-independent and Pluripotent State-Dependent Glycogen Synthesis Mediated by GSK3i and Naïve Growth Conditions in hPSCs

Interestingly, pharmacological inhibition of GSK-3 induces the formation of glycogen bodies when hPSCs were grown and assayed on the plastic coverslip (Fig 3). Moreover, the formation of the inclusion of glycogen bodies mediated by GSK3i was different from that of BMP-4, because the formation of glycogen bodies was independent of the pluripotent cell state of hPSCs (Fig 4). These data may also suggest that GSK3i might stimulate the transition from the primed to the naïve state in hPSCs, rather than inducing specific lineage differentiation. The formation of glycogen bodies together with sustained Oct-4 expression depicts this dynamic change of the altered pluripotent state.

GSK-3 is a potent regulator of the Wnt-β-catenin pathway. GSK-3 inhibits the Wnt signaling pathway by phosphorylating β-catenin and indirectly mediating its proteasomal degradation [39]. However, the roles of GSK-3 in the regulation of the pluripotent states of embryonic stem cell culture are controversial. An initial report suggests that GSK-3 inhibition sustains the pluripotency and self-renewal in both mESCs and hESCs [40]. However, a recent study indicates that GSK-3 inhibition-mediated activation of the Wnt- β-catenin pathway actually promotes hESC differentiation, rather than sustaining hESC pluripotency and self-renewal [41]. Perhaps, GSK-3 inhibition is only required for supporting the naïve pluripotent state in mESCs, but not that primed pluripotent state in hESCs. Indeed, the use of two small-molecule inhibitors (2i) (that included GSK3i and MEKi) in the presence of LIF support the naïve state in mESCs [14].

**Fig 9:**
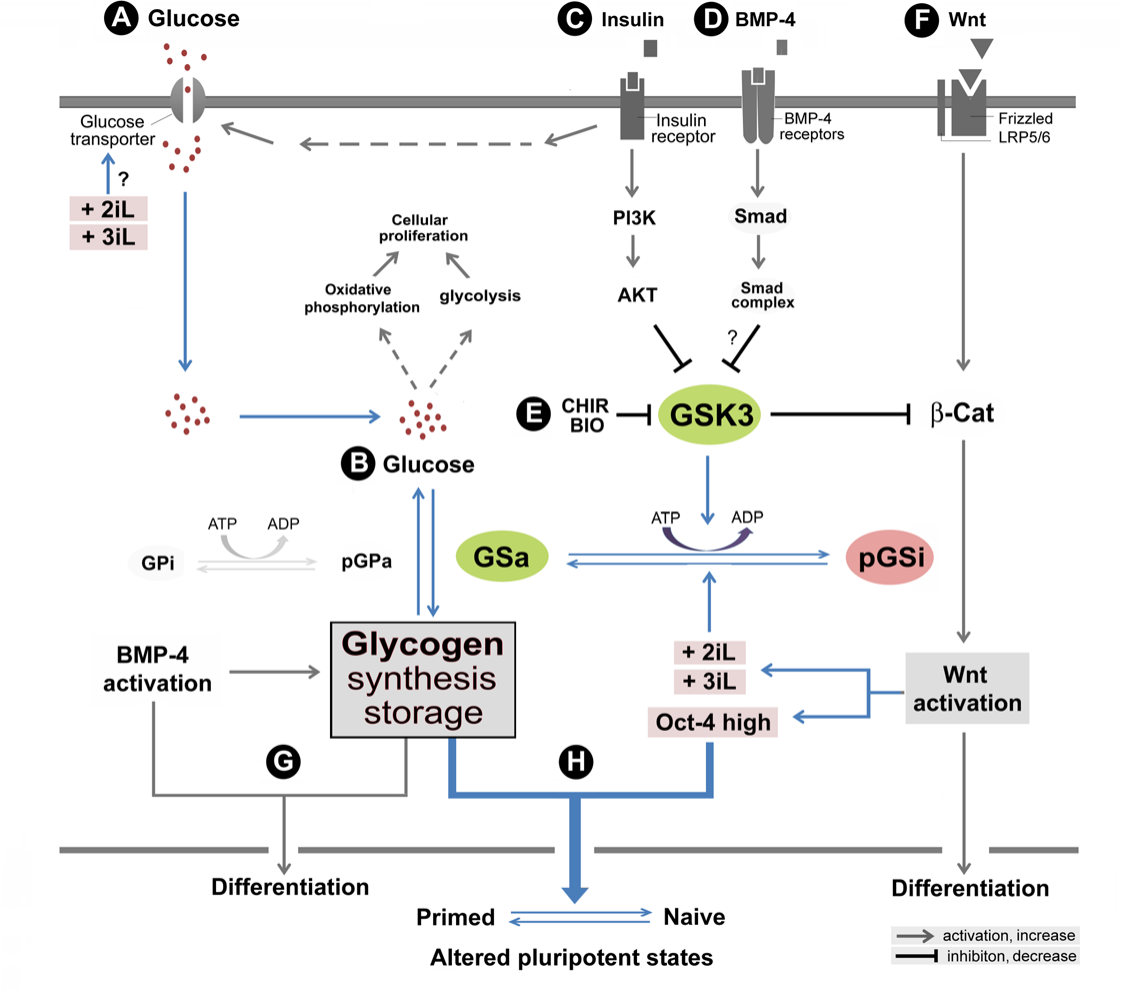
Growth, differentiation, and glycogen synthesis in human embryonic stem cells (hESCs). A hypothetical model is presented to elucidate major signaling pathways that are associated with glycogen synthase kinase 3 (GSK-3) and glycogen synthesis. (**A)** In this model, glucose transporter-mediated uptake of glucose is activated by an insulin-receptor signaling pathway. (**B**) Glucose takes part in aerobic glycolysis in the cytoplasm and oxidative phosphorylation in mitochondria to produce energy for hESC proliferation and self-renewal. Presumably, excessive glucose is converted to glycogen by activated glycogen synthase (GSa) upon stress and differentiation signaling to enhance hPSC survival. Glycogen can be decomposed in the presence of phosphorylated glycogen phorsphoylase (pGP) whenever necessary. (**C**) The insulin signaling pathway also activates the PI3K-AKT pathway, which phosphorylates GSK-3. The GSK-3 phosphorylation leads to its inactivation and subsequently inhibits the phosphorylation of glycogen synthase (GS). Thus, activation of the PI3K-AKT pathway increases glycogen synthesis. (**D**) The mechanism of BMP-4-induced glycogen body formation is likely through the inhibition of GSK-3 by the putative Smad pathways. (**E**) The mechanism by which the GSK3i CHIR modulates the synthesis of glycogen is likely through the inhibition of GSK-3 activity, thereby altering glycogen synthase activity. (**F**) Concomitantly, GSK-3 inhibitors (e.g., CHIR99021 and BIO) may promote hPSC differentiation by activation of the β-catenin-WNT pathway. (**G**) The function of aggregated glycogen bodies is unclear and may be associated with response to extracellular stress and differentiation signals such as BMP-4. (**H**) Under sustained Oct-4 expression conditions, GSK3i-mediated glycogen accumulation concomitant with Wnt activation and other naïve growth components enhances the transition from the primed pluripotent to the naïve state in hPSCs. The proposed mechanisms in this model supported by this study are color-highlighted. The “?” symbols indicate inconclusive observations. The abbreviations are: 2iL, the naïve pluripotent growth condition that include GSK3i, MEKi, and LIF; 3iL, the naïve pluripotent growth condition that include GSK3i, MEKi, BMP4i, and LIF; AKT, the serine-threonine protein kinase encoded by v-akt murine thymoma viral oncogene homolog; CHIR, CHIR99021; GPi, dephosphorylated glycogen phosphorylase (inactive form); GSa, dephosphorylated glycogen synthase (active form); GSK-3, glycogen synthase kinase 3; pGPa, phosphorylated glycogen phosphorylase (active form); pGSi, phosphorylated glycogen synthase (inactive form); PI3K, the phosphoinositide 3-kinase; and β-cat, β-catenin.

Furthermore, using naïve human stem cell medium (NHSM) that contains LIF and 2i, Hanna and colleagues were also able to directly convert primed hPSCs to the naïve state [15]. Based on 2i and LIF, several research groups have successfully developed different protocols for direct conversion of primed hPSCs to the naïve state [13, 16–18]. We used a naïve hPSC culture protocol based on the 3iL [13], which was compatible with our current culture platforms. We were able to systematically address glycogen variations under different naïve conditions (e.g., BIO, 2i, 3i, 2iL, and 3iL). Our data suggest that primed hPSCs treated with naïve growth conditions acquire altered pluripotent states, similar to those naïve-like hPSCs, with increased glycogen synthesis (Figs 5–8). It appears that GSK3i is a necessary, but insufficient element, for the regulation of the distinct pluripotent states. However, it is the essential component to trigger the naïve state associated glycogen synthesis. The addition of more naïve components such as MEKi and LIF in the naïve growth protocols might play a role in stabilizing the metabolic state that are associated with the naïve pluripotency (Fig 9).

### Regulation of Glycogen Synthesis and Functional Implications

It is suggested that the equilibrium between glucose utilization and glycogen synthesis reflects the energetic states of cells, which are regulated by glycogen phosphorylase and glycogen synthase respectively [42] (Fig 9). This equilibrium is further controlled by the insulin-signaling-activated PI3K-AKT pathway. In this regard, AKT is shown to promote glycogen synthesis by phosphorylating/inactivating GSK-3 [43, 44], which in turn activates glycogen synthase by inhibiting its phosphorylation (Fig 9). Thus, the mechanism by which GSK-3 inhibitors (e.g., CHIR99021 and BIO) enhance glycogen synthesis and subsequent glycogen body formation in hPSCs might be homologous to that of AKT. Clearly, the connections of these networks might ensure that the metabolism of hPSCs is coordinately regulated by a general mechanism when the cells receive different stimuli (Fig 9).

The potential molecular mechanisms that regulate glycogen synthesis are likely related to the major phosphorylation residue (Ser641) of GS (Fig 8F–8H), Other unidentified phosphorylation sites may be also involved in this regulation. Future experiments could be performed to define the role of various phosphorylation sites of glycogen synthase in the regulation of glycogen synthesis. Furthermore, genetic experiments using expression vectors that bear various dominant negative mutants of both GSK-3α and GSK-3β should provide direct evidence regarding the contribution of GSK-3 to the regulation of glycogen synthesis and the formation of glycogen bodies. In addition, GS-independent pathways that involve mitochondrial glucose oxidation and mitochondrial biogenesis might be an important area to be investigated [21, 45, 46].

The functions of increased glycogen synthesis and storage in hPSCs are poorly understood. Physiologically, glycogen storage is mainly present in liver and muscles and used for glycogenolysis on demand. Glycogen content varies in different cancer cell lines, ranging from 0 to 0.25 μg per μg proteins [24, 33]. The levels of glycogen were demonstrated to be particularly high in breast, kidney, uterus, bladder, ovary, skin, and brain cancer cell lines. Glycogen content in these cells was inversely correlated with the proliferation rate [47]. Recent studies showed a glycogen phosphorylase inhibitor could inhibit cancer growth and trigger apoptosis in pancreatic cancer cells, likely through restraining glycogen breakdown and subsequent glucose oxidation [48]. Glycogen phosphorylase depletion induced glycogen accumulation also prompts premature senescence and impairs tumor growth via a reactive oxygen species (ROS)-dependent mechanism [49]. In contrast, other studies indicated that glycogen accumulation were associated with cancer cell survival under glucose deprivation and hypoxic environments [24, 50]. These data reveal distinct cancer cell fates under glycogen accumulation, which may be regulated in cell-type-, growth environment-, and temporal and spatial-dependent manners.

In the case of hPSCs, for example, primed H1 colonies cultured on MEF had 0.28 μg glycogen per μg proteins, 7-fold higher than MCF7 cells (Fig 1). Apparently, the glycogen content was increased in hPSCs cell grown on Matrigel (Fig 1), suggesting extracellular matrices and growth conditions may have greater influence on glycogen accumulation. Possibly, altered glycogen synthesis serves as an acute stress-response sensor that may be important for self-renewal, cell survival, and differentiation under various growth conditions. Dynamic changes of glycogen synthesis in distinct pluripotent states reflect developmental restrains and entries for glycolysis, oxidation respiration, and glycogen metabolisms.

In summary, our findings indicate that glycogen synthesis was active in hPSCs, which is regulated by BMP-4 and GSK-3-related pathways. Glycogen body formation and glycogen utilization could potentially be useful assays to define cellular heterogeneity, the pluripotent and energetic states of hPSCs. Our study provides molecular clues concerning how glycogen synthesis is regulated in hPSCs under various naïve growth conditions. It is possible that the equilibrium between glycogen accumulation and glycogenolysis represents a metabolic switch to control glycolytic states in hPSCs, which in turn regulate in naïve and primed states. Thus, glycogen metabolism might regulate pluripotent state transitions and cellular differentiation in hPSCs.

## Supporting Information

S1 Fig TEM analysis of glycogen synthesis and the formation of glycogen bodies mediated by GSK-3 inhibition in H1 hESCs. Glycogen synthesis in untreated H1 cells (control, **A**-**E**) and in 3 μM GSK3i (CHIR99021)-treated H1 cells (**F**-**I**) grown on Matrigel-coated plastic coverslips as described in Materials and Methods. The annotations in the TEM graphs were indicated by red-colored arrowheads. The asterisk signs (in TEM graphs) indicate glycogen defect regions in the glycogen body, which likely resulted from dissociation of glycogen aggregates when the specimens were floating in solution during sample preparation. Abbreviations: G, various sizes of glycogen aggregates; GB, glycogen bodies with defined boundaries; PM, plasma membrane; M, mitochondria; NM, nuclear membrane; Nu, the nucleus of the cells; Nuo, the nucleolus. Scale bars were indicated in each graph.
(PDF)

S2 Fig TEM analysis of glycogen synthesis and the formation of glycogen bodies mediated by GSK-3 inhibition in BC1 iPSCs. Glycogen synthesis in untreated **BC1** cells (control, **A**-**D**) and in 3 μM GSK3i (CHIR99021)-treated H1 cells (**E**-**H**) grown on Matrigel-coated plastic coverslips as described in Materials and Methods. The legends and abbreviations to S2 Fig are as described for S1 Fig.
(PDF)
