# Supplementary material for: Variations in Glycogen Synthesis in Human Pluripotent Stem Cells with Altered Pluripotent States
Source: PLoS One. 2015 Nov 13;10(11):e0142554. doi: 10.1371/journal.pone.0142554 (PMC4643957; doi:10.1371/journal.pone.0142554)
Supplement: S1 Fig — Glycogen synthesis in untreated H1 cells (control, A-E) and in 3 μM GSK3i (CHIR99021)-treated H1 cells (F-I) grown on Matrigel-coated plastic coverslips as described in Materials and Methods. The annotations in the TEM graphs were indicated by red-colored arrowheads. The asterisk signs (in TEM graphs) indicate glycogen defect regions in the glycogen body, which likely resulted from dissociation of glycogen aggregates when the specimens were floating in solution during sample preparation. Abbreviations: G, various sizes of glycogen aggregates; GB, glycogen bodies with defined boundaries; PM, plasma membrane; M, mitochondria; NM, nuclear membrane; Nu, the nucleus of the cells; Nuo, the nucleolus. Scale bars were indicated in each graph. (PDF) [file pone.0142554.s001.pdf]

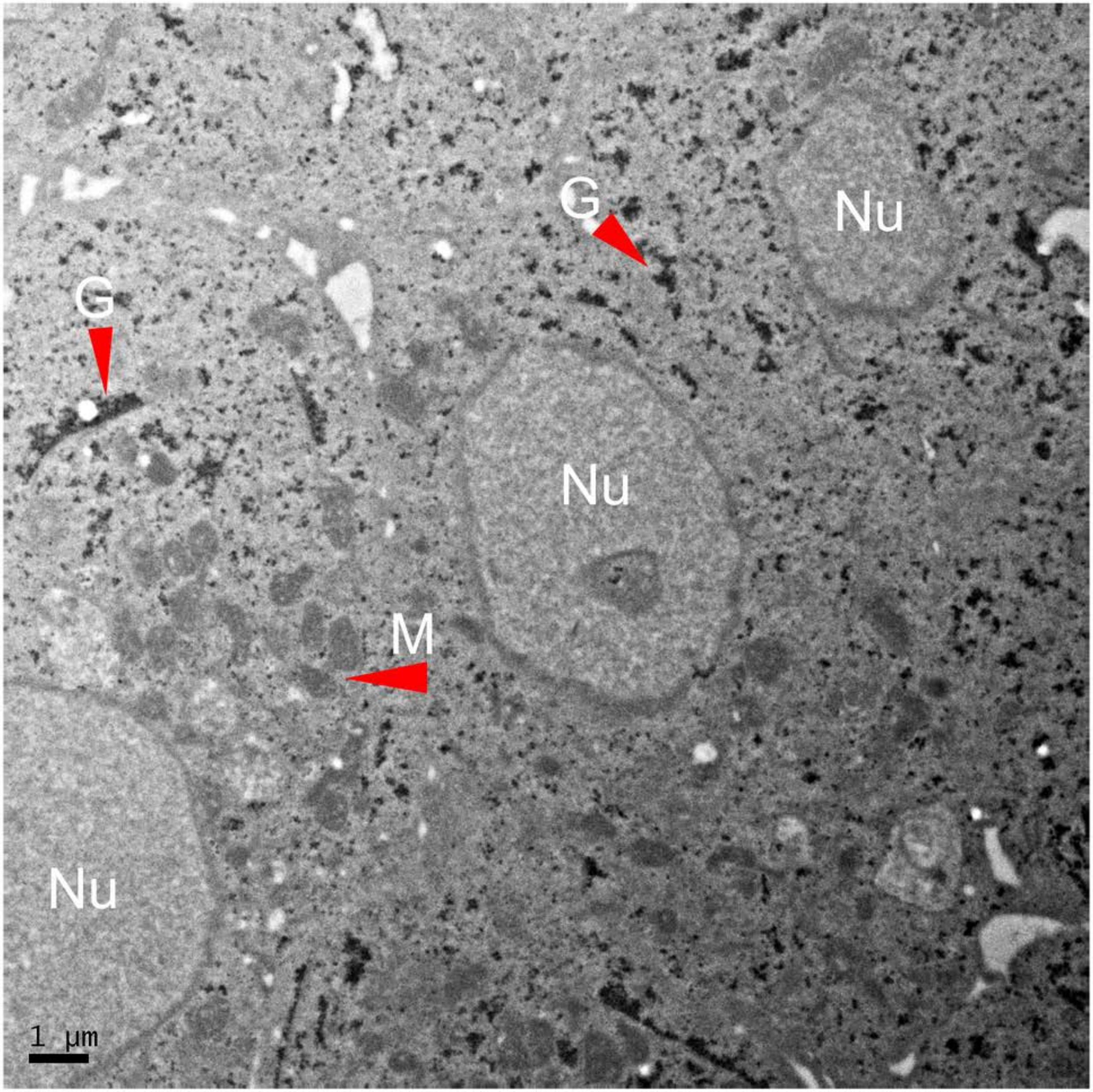

S1 Fig. (A) TEM analysis of glycogen synthesis and the formation of glycogen bodies in untreated H1 hESCs (control)

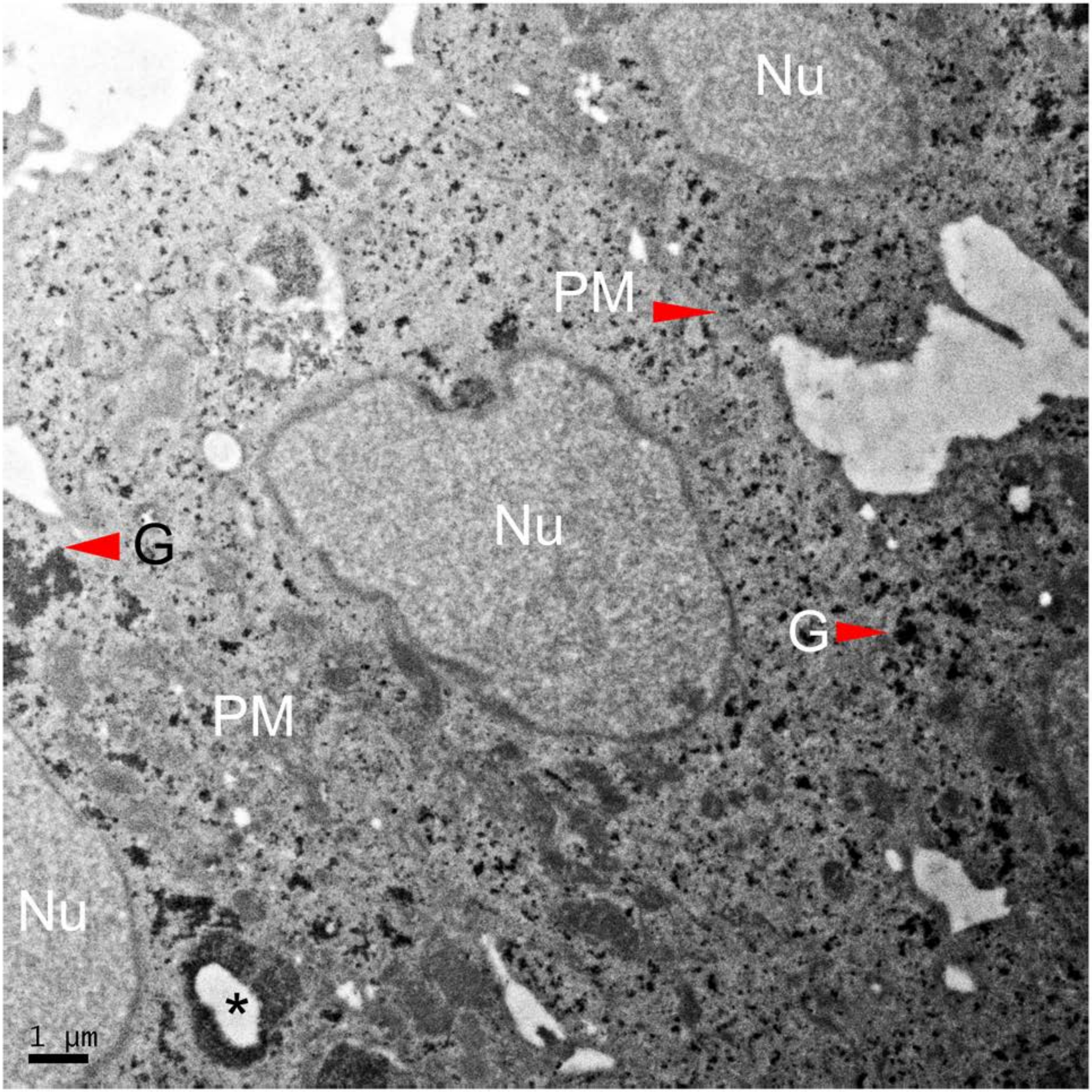

S1 Fig. (B) TEM analysis of glycogen synthesis and the formation of glycogen bodies in untreated H1 hESCs

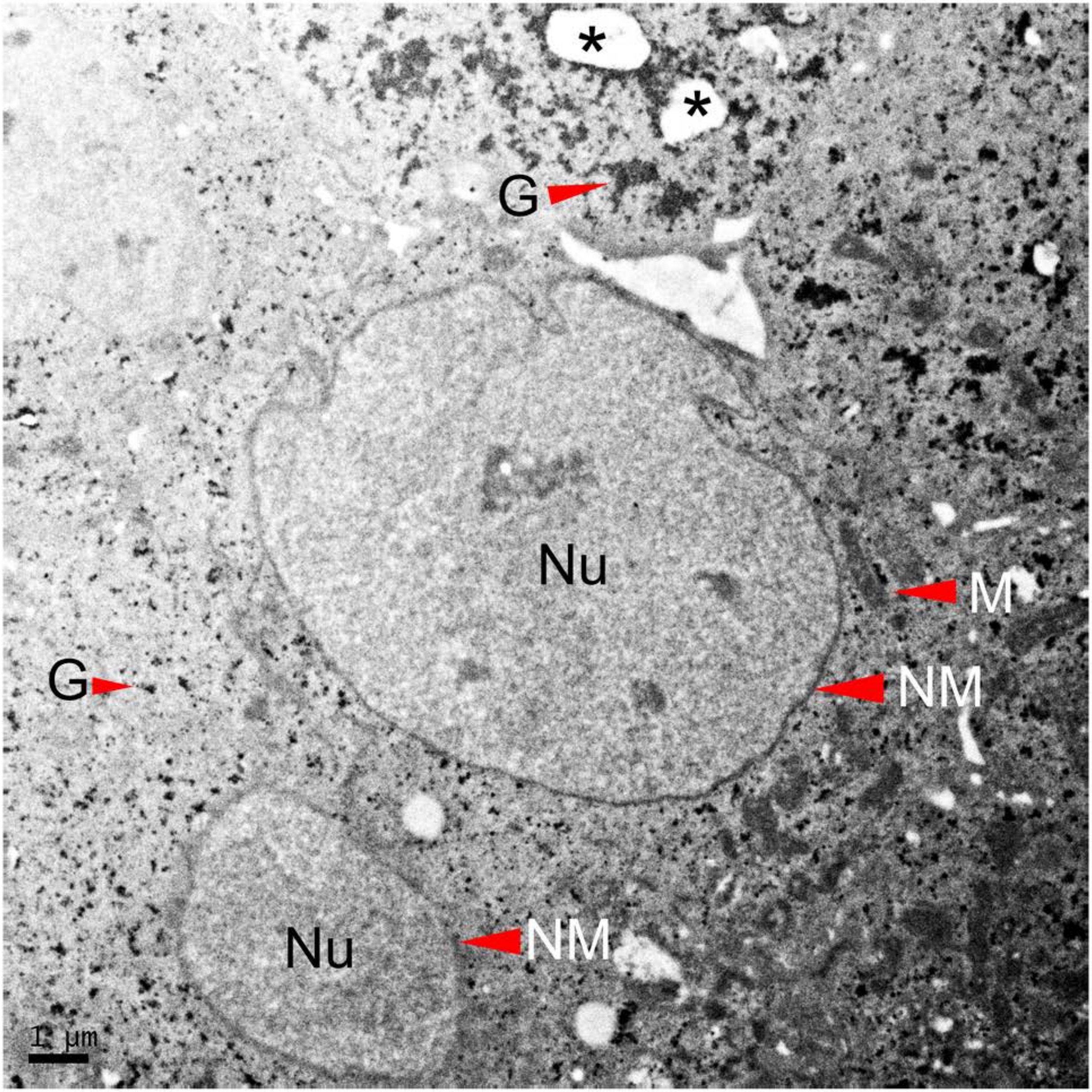

S1 Fig. (C) TEM analysis of glycogen synthesis and the formation of glycogen bodies in untreated H1 hESCs

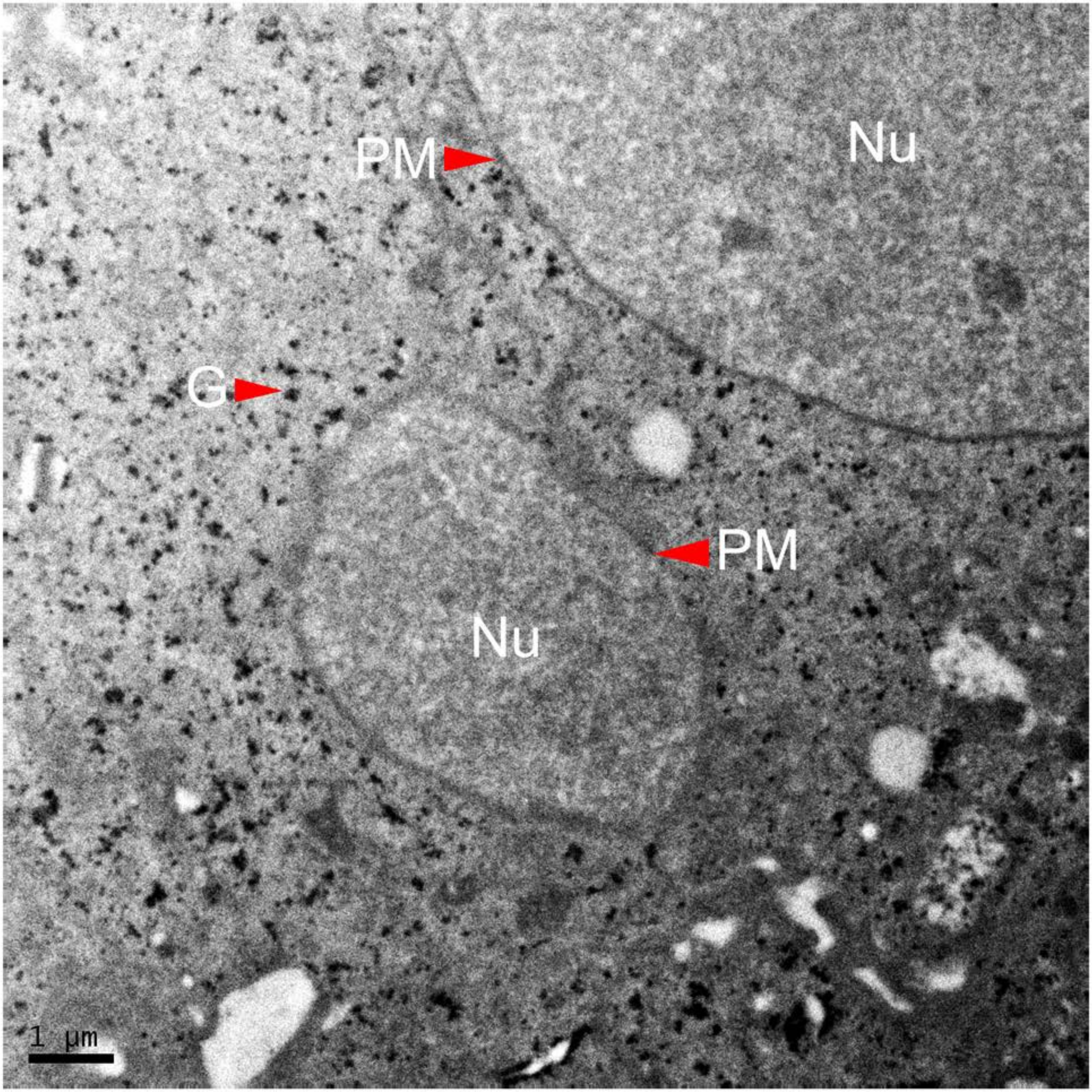

S1 Fig. (D) TEM analysis of glycogen synthesis and the formation of glycogen bodies in untreated H1 hESCs

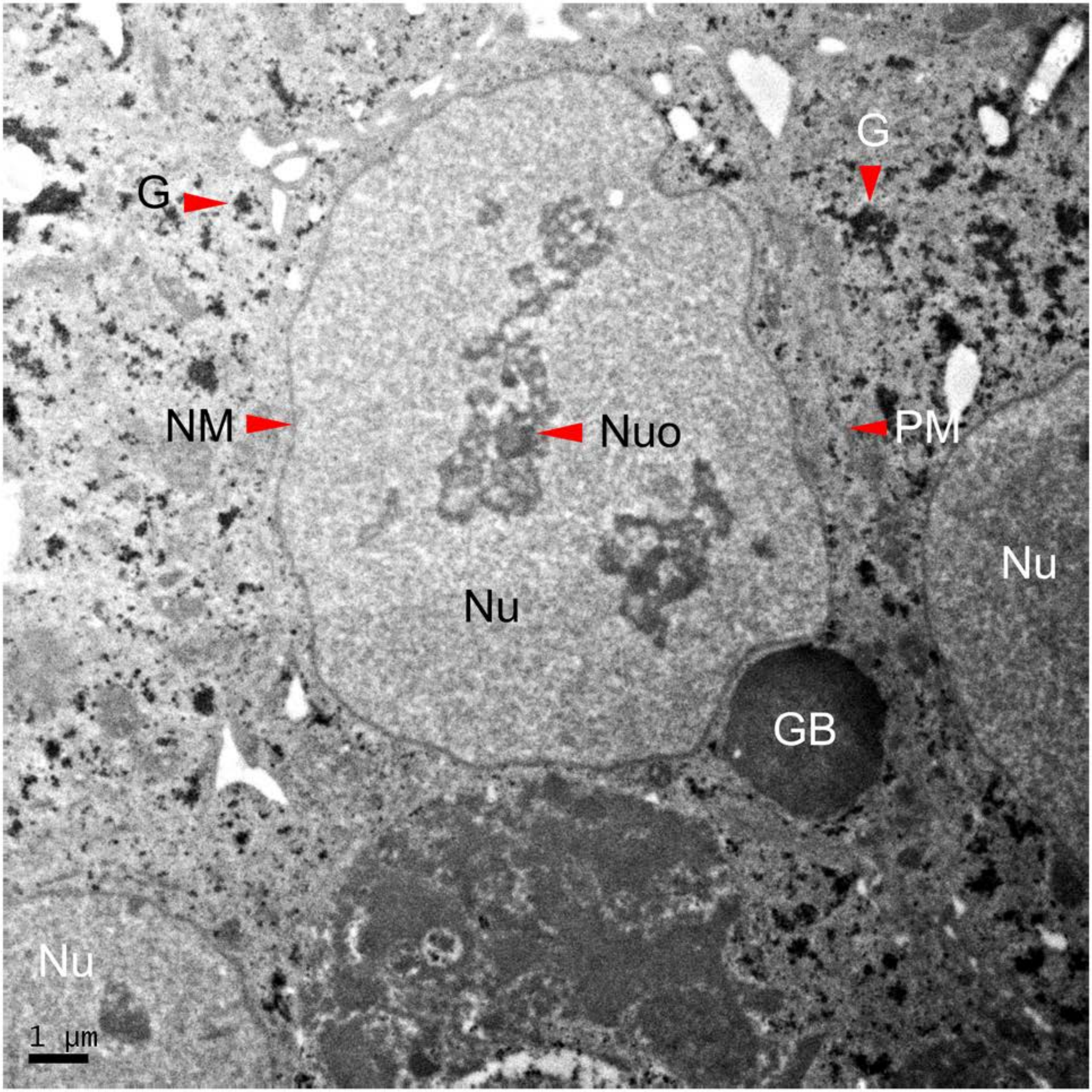

S1 Fig. (E) TEM analysis of glycogen synthesis and the formation of glycogen bodies in untreated H1 hESCs (control)

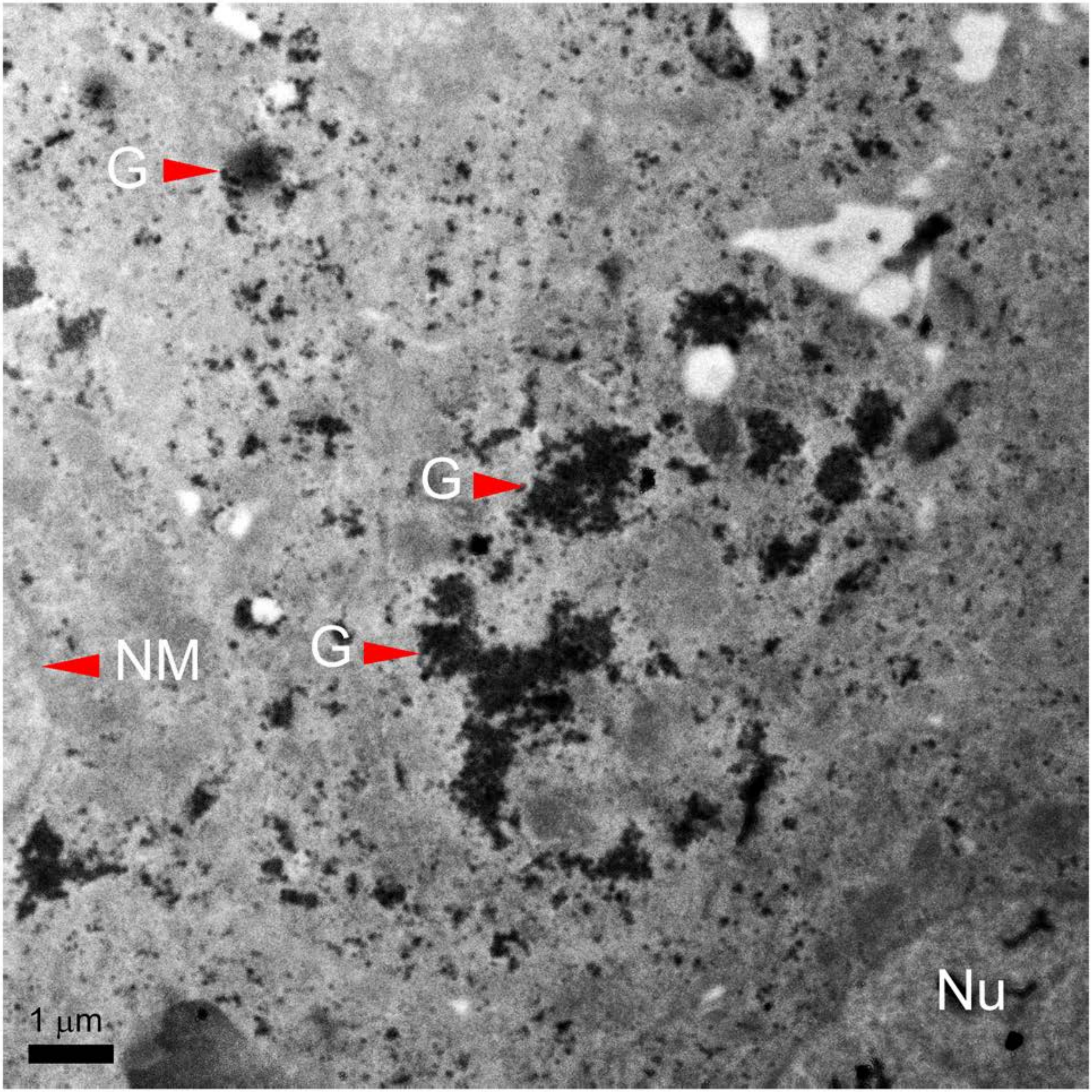

S1 Fig. (F) TEM analysis of glycogen synthesis and the formation of glycogen bodies mediated by GSK-3 inhibition in H1 hESCs

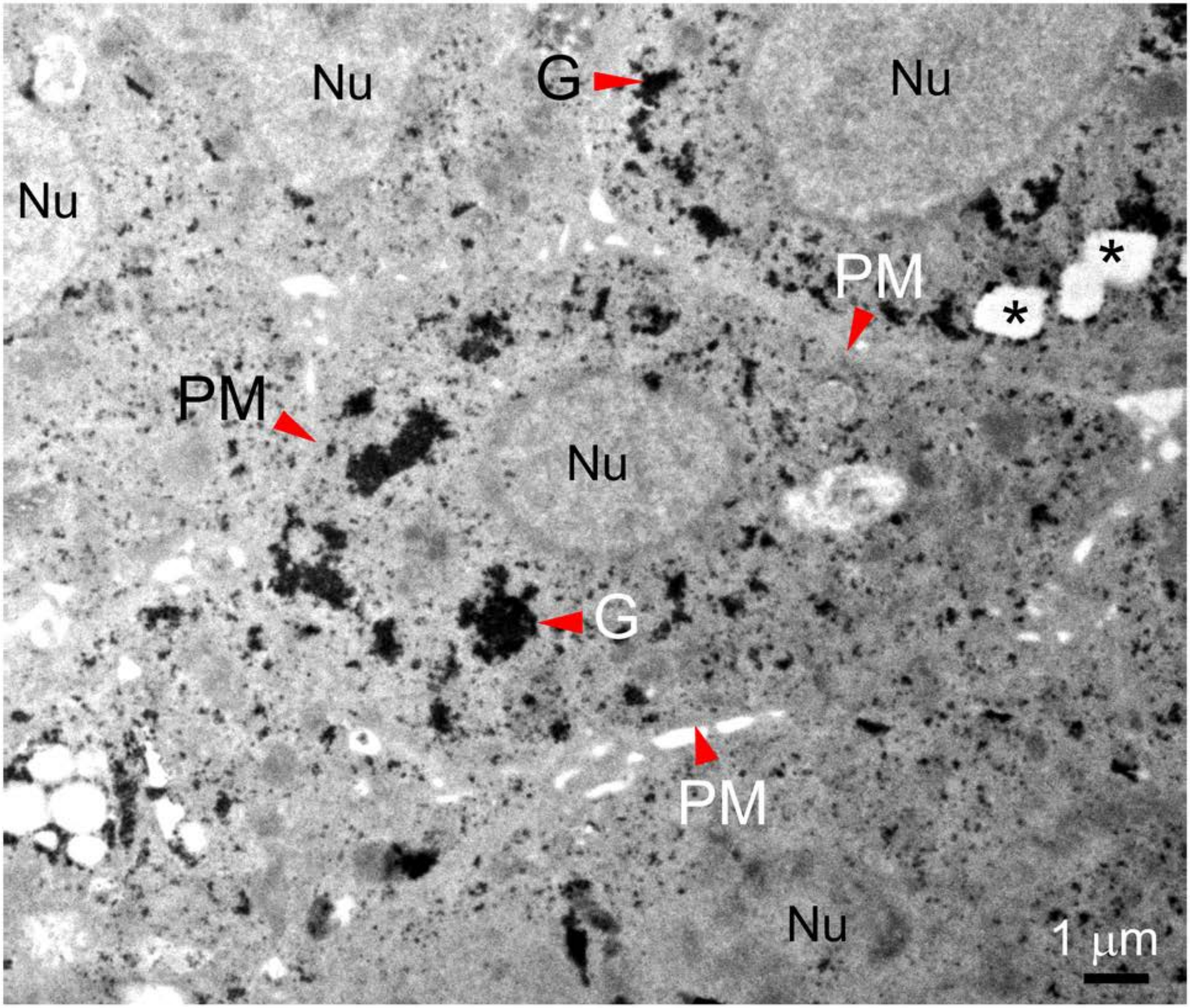

S1 Fig. (G) TEM analysis of glycogen synthesis and the formation of glycogen bodies mediated by GSK-3 inhibition in H1 hESCs.

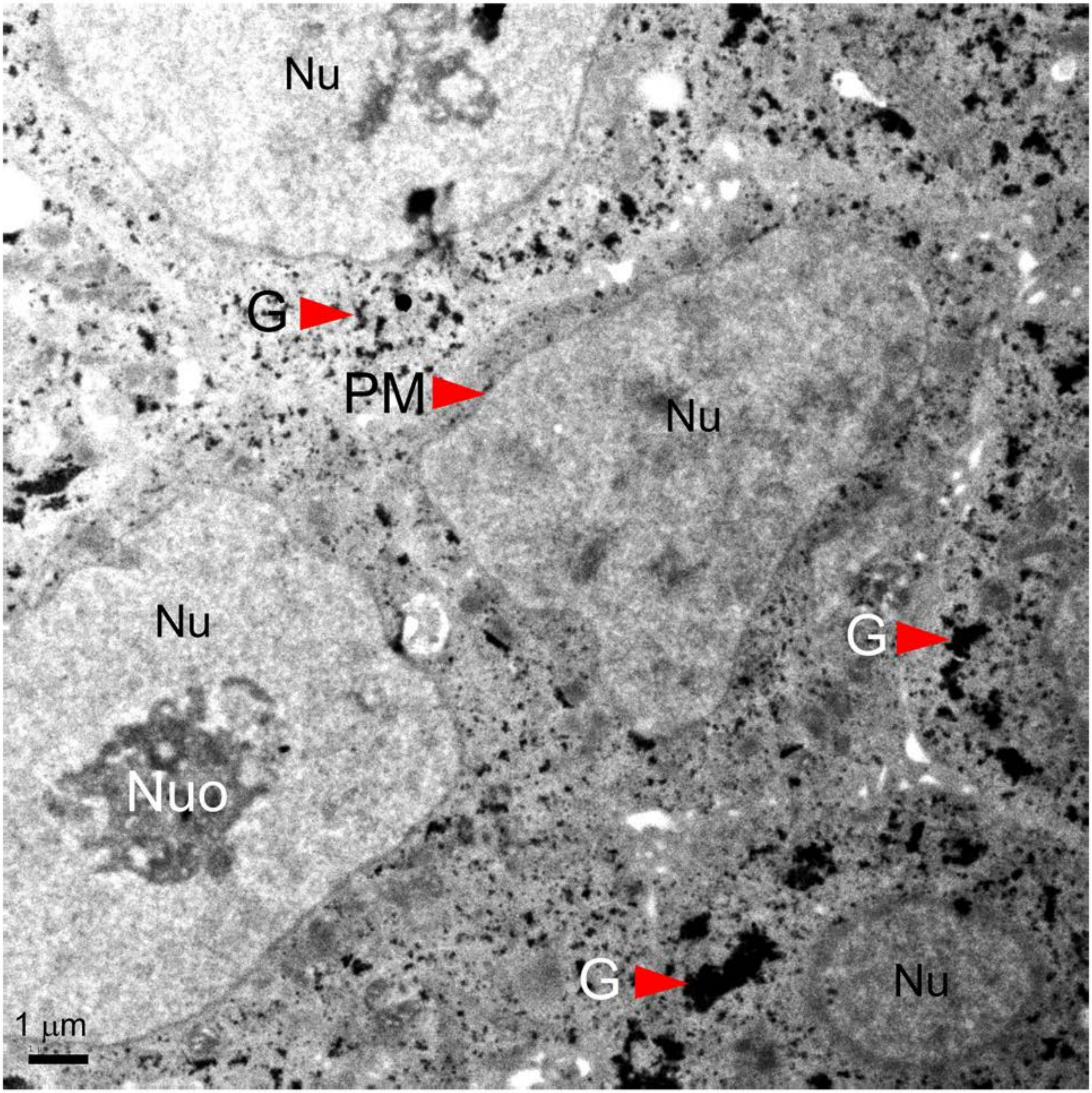

S1 Fig. (H) TEM analysis of glycogen synthesis and the formation of glycogen bodies mediated by GSK-3 inhibition in H1 hESCs.

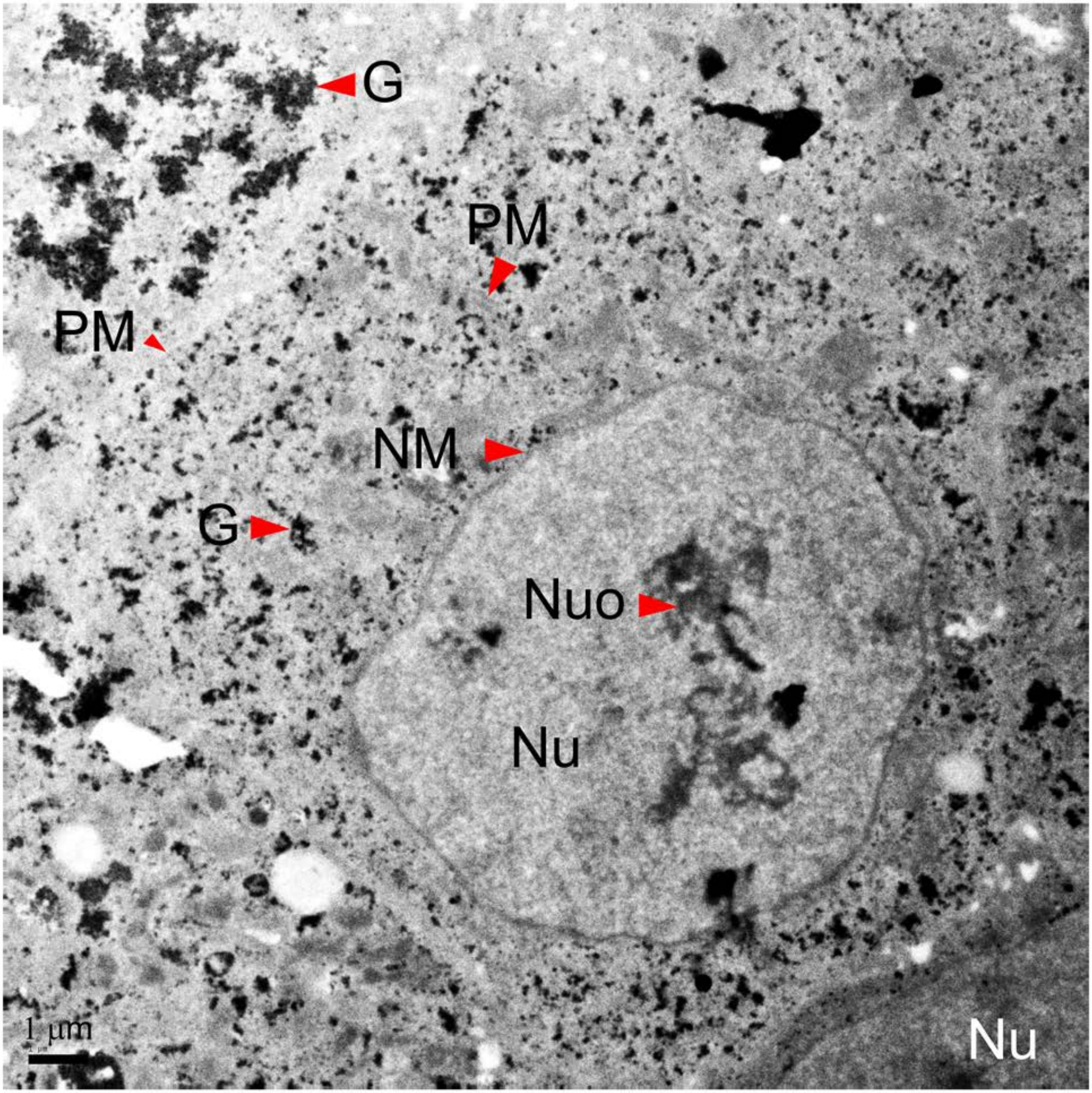

S1 Fig. (Ⅰ) TEM analysis of glycogen synthesis and the formation of glycogen bodies mediated by GSK-3 inhibition in H1 hESCs.
