# Supplementary material for: Variations in Glycogen Synthesis in Human Pluripotent Stem Cells with Altered Pluripotent States
Source: PLoS One. 2015 Nov 13;10(11):e0142554. doi: 10.1371/journal.pone.0142554 (PMC4643957; doi:10.1371/journal.pone.0142554)
Supplement: S2 Fig — Glycogen synthesis in untreated BC1 cells (control, A-D) and in 3 μM GSK3i (CHIR99021)-treated H1 cells (E-H) grown on Matrigel-coated plastic coverslips as described in Materials and Methods. The legends and abbreviations to S2 Fig are as described for S1 Fig. (PDF) [file pone.0142554.s002.pdf]

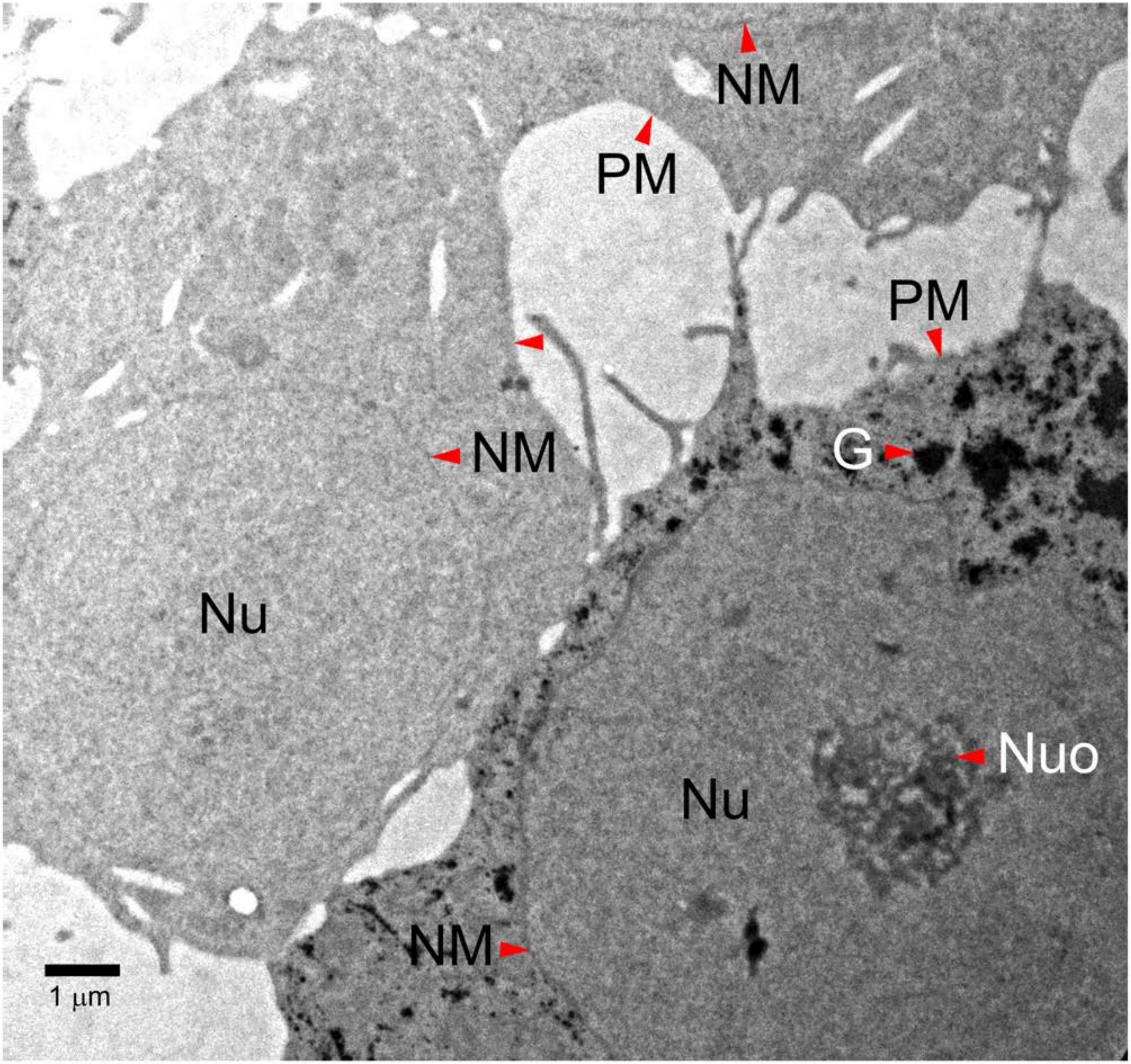

S2 Fig. (A) TEM analysis of glycogen synthesis and the formation of glycogen bodies in untreated BC1 iPSCs

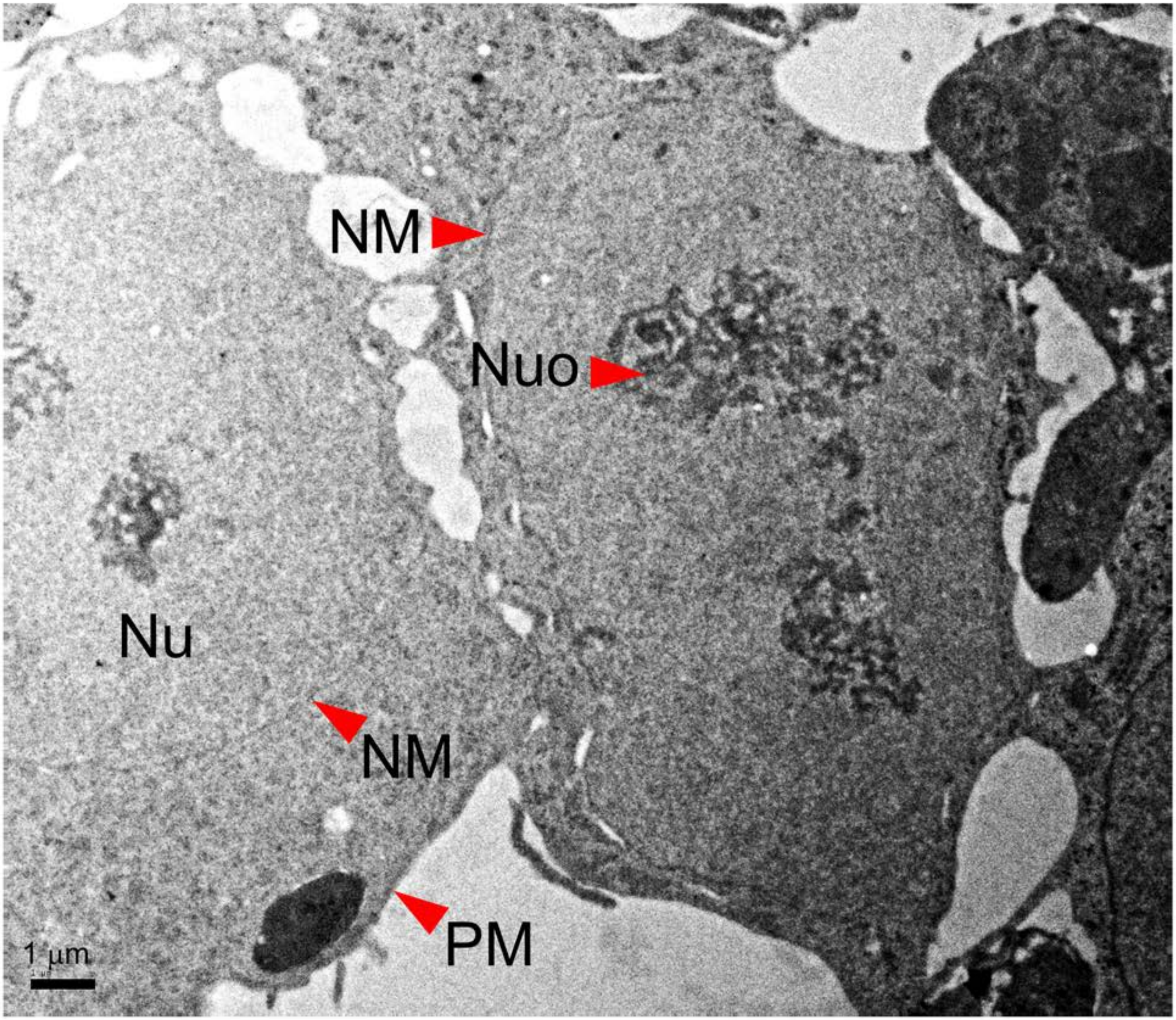

S2 Fig. (B) TEM analysis of glycogen synthesis and the formation of glycogen bodies in untreated BC1 iPSCs

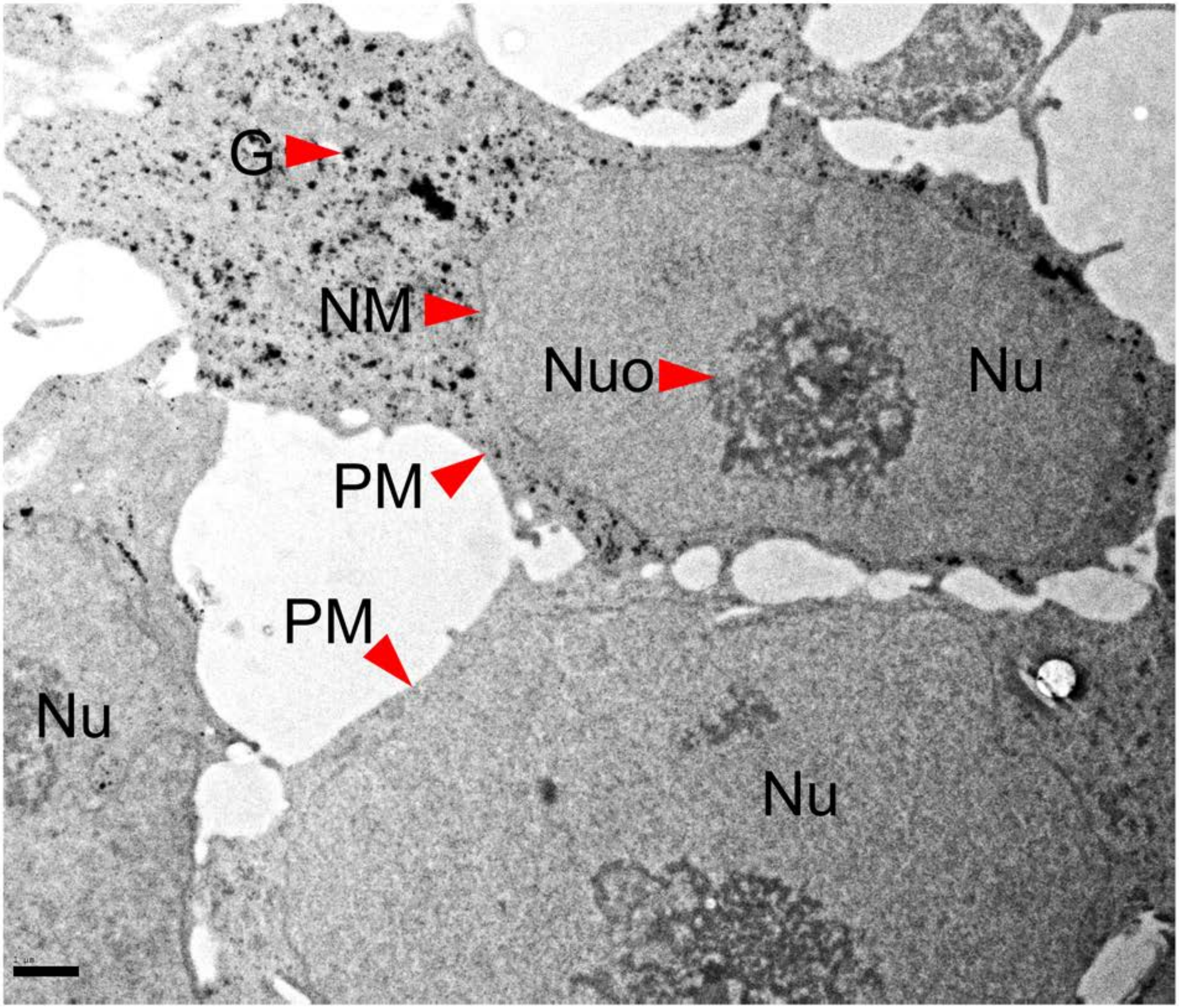

S2 Fig. (C) TEM analysis of glycogen synthesis and the formation of glycogen bodies in untreated BC1 iPSCs

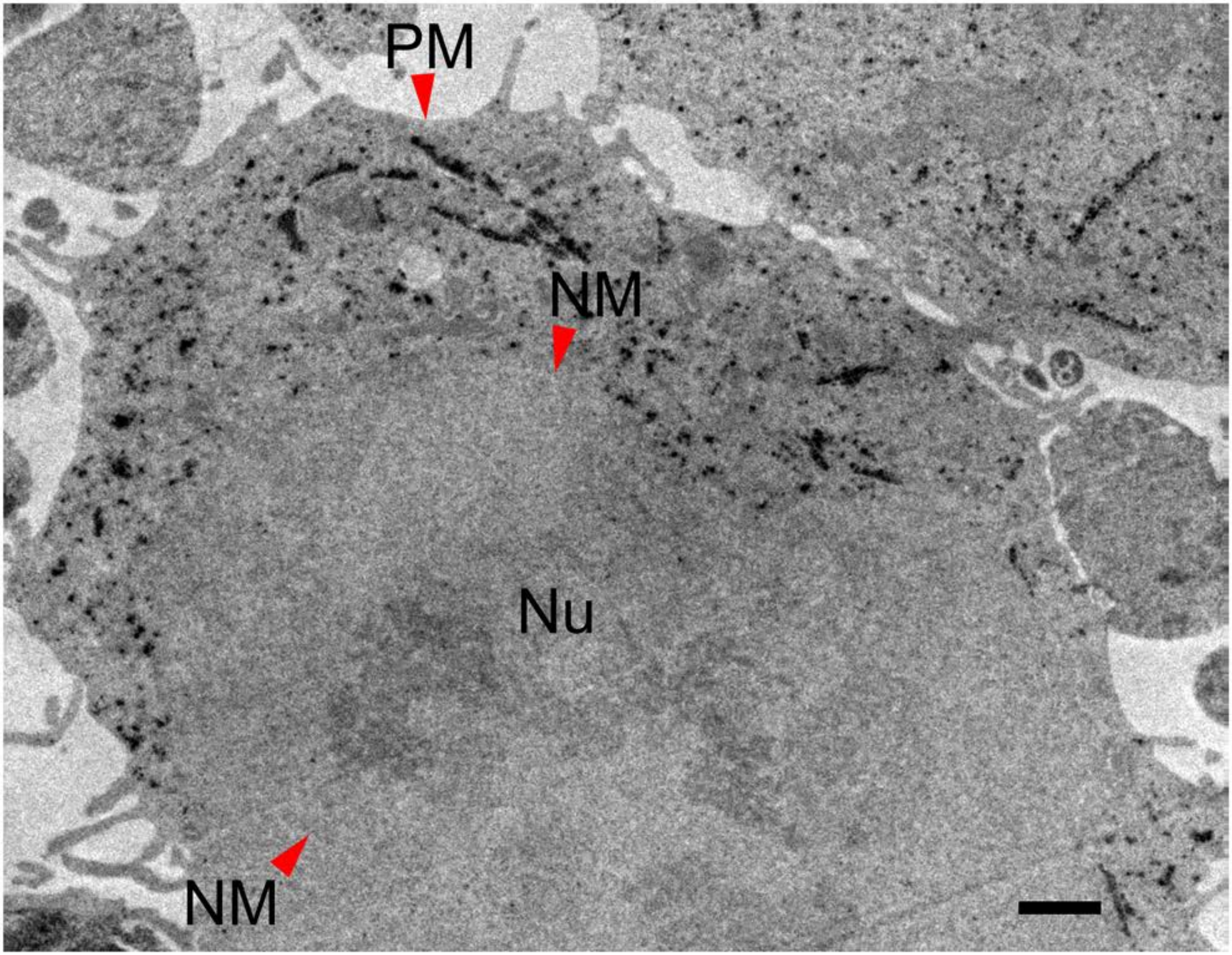

S2 Fig. (D) TEM analysis of glycogen synthesis and the formation of glycogen bodies in untreated BC1 iPSCs

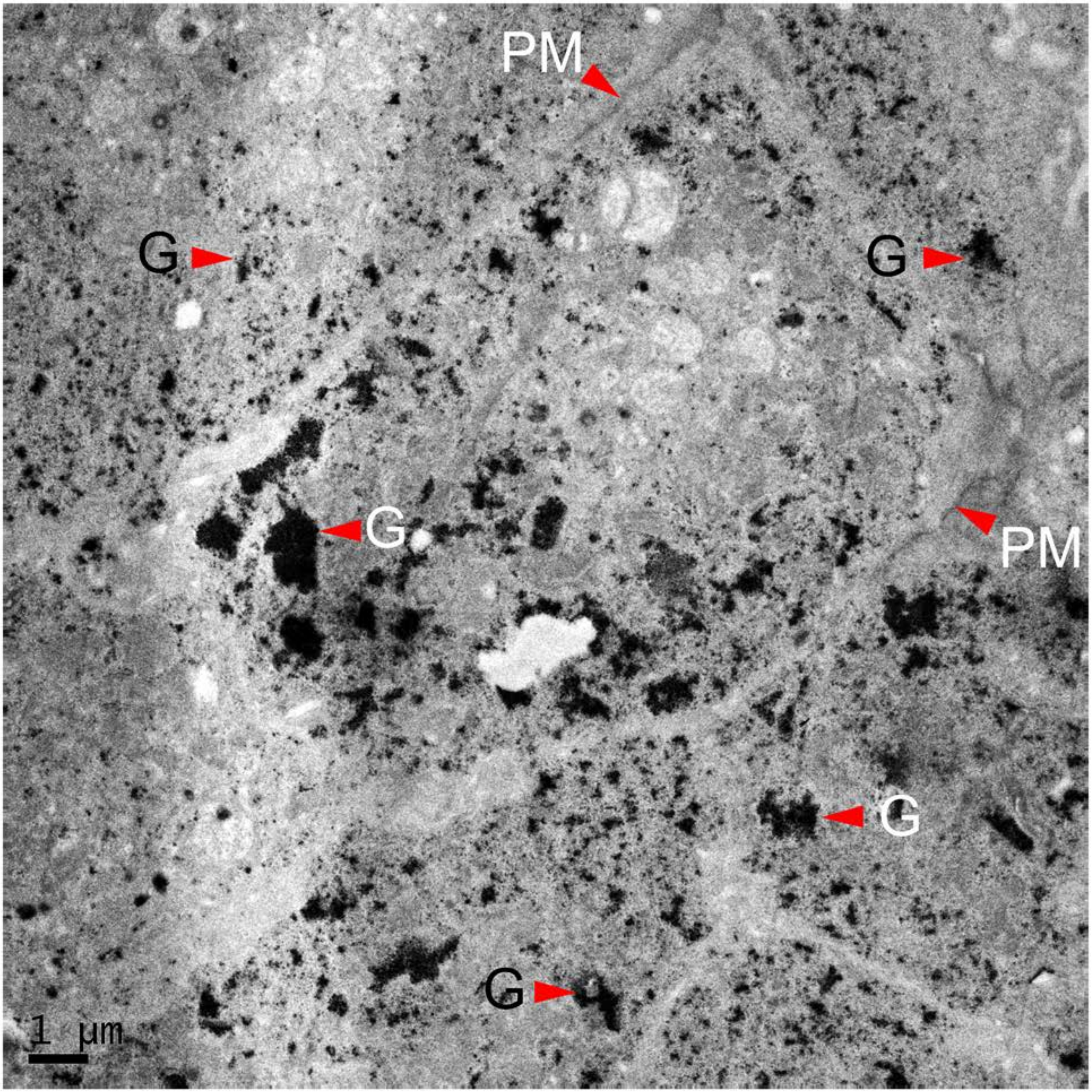

S2 Fig. (E) TEM analysis of glycogen synthesis and the formation of glycogen bodies mediated by GSK-3 inhibition in BC1 iPSCs.

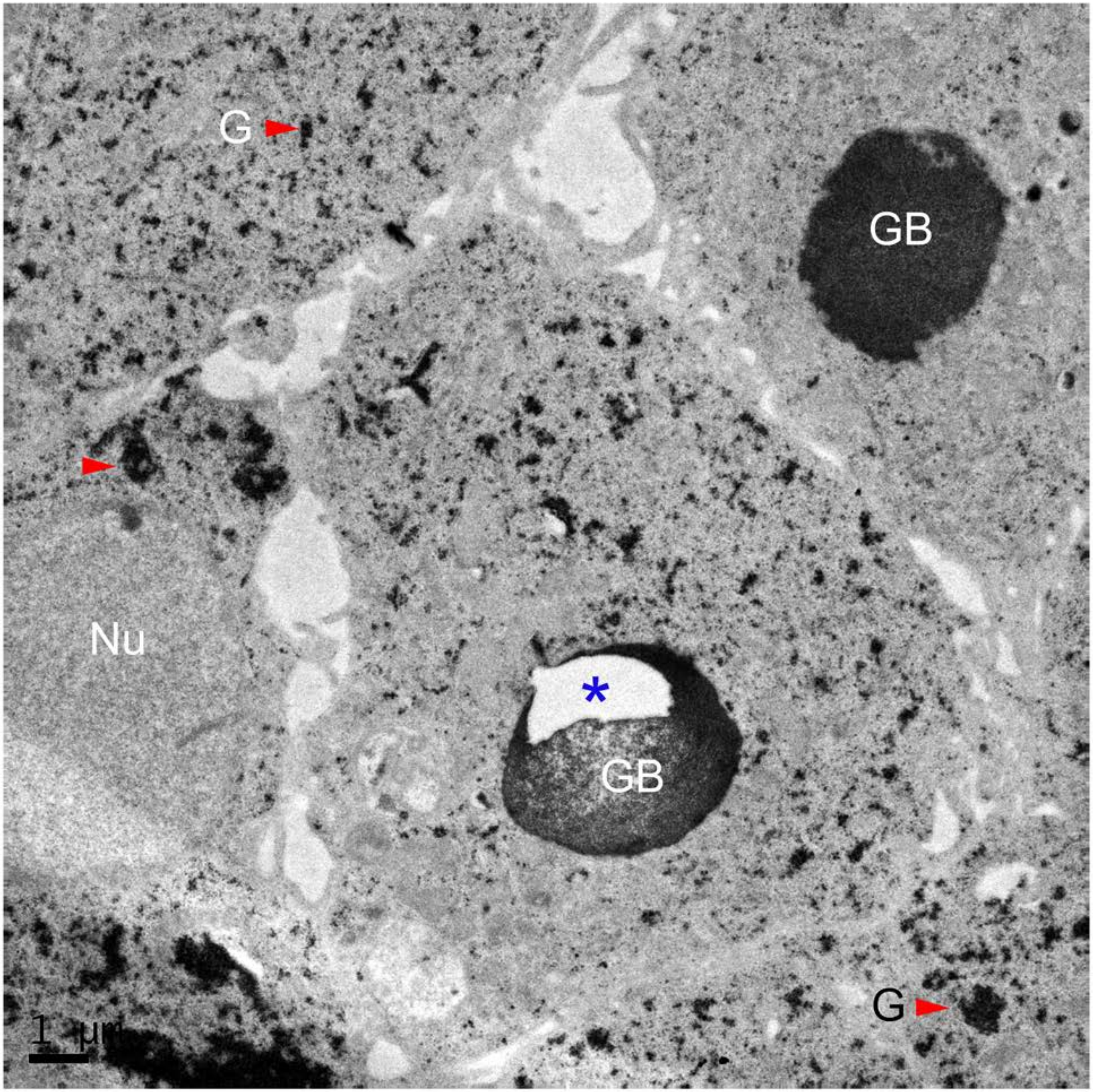

S2 Fig. (F) TEM analysis of glycogen synthesis and the formation of glycogen bodies mediated by GSK-3 inhibition in BC1 iPSCs.

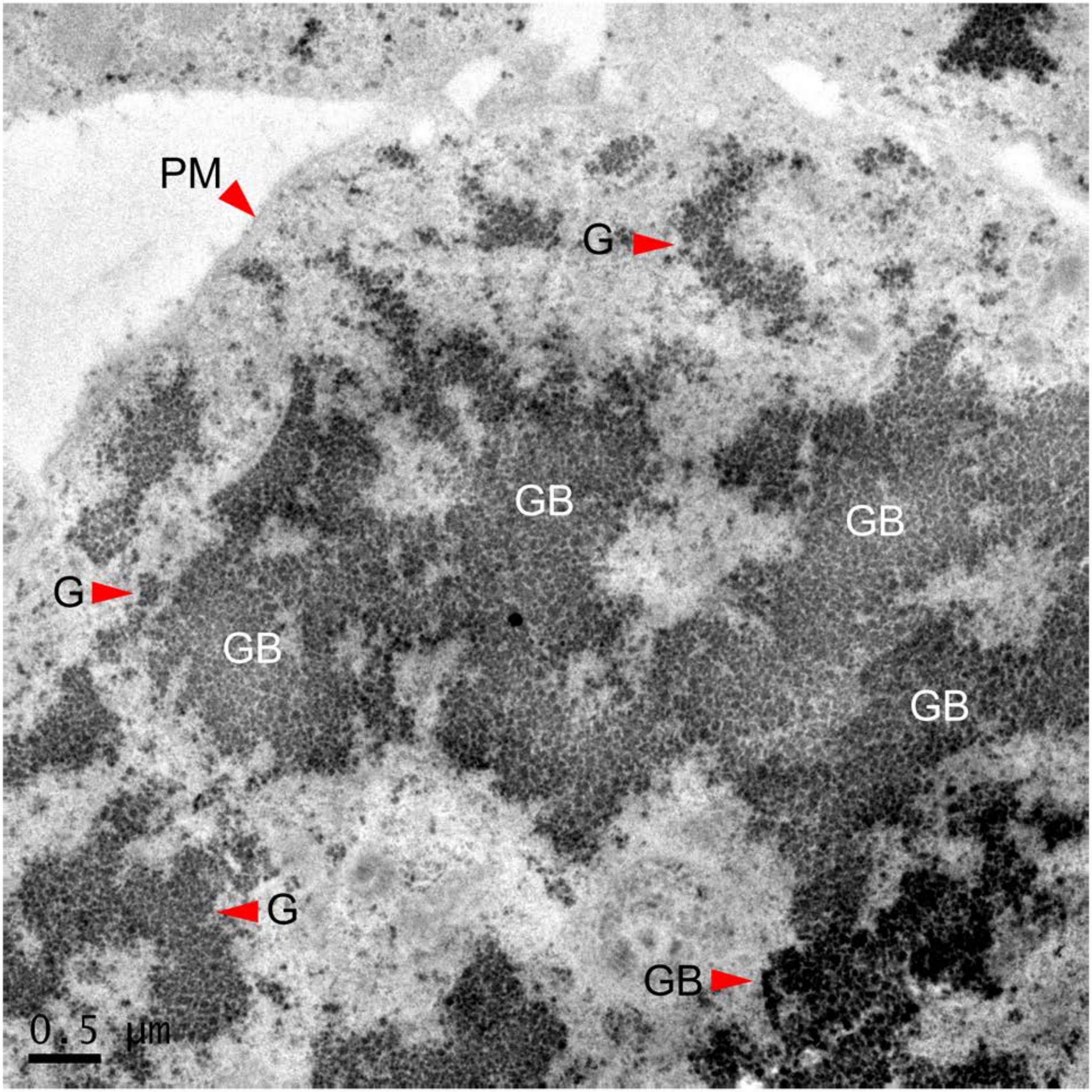

S2 Fig. (G) TEM analysis of glycogen synthesis and the formation of glycogen bodies mediated by GSK-3 inhibition in BC1 iPSCs.

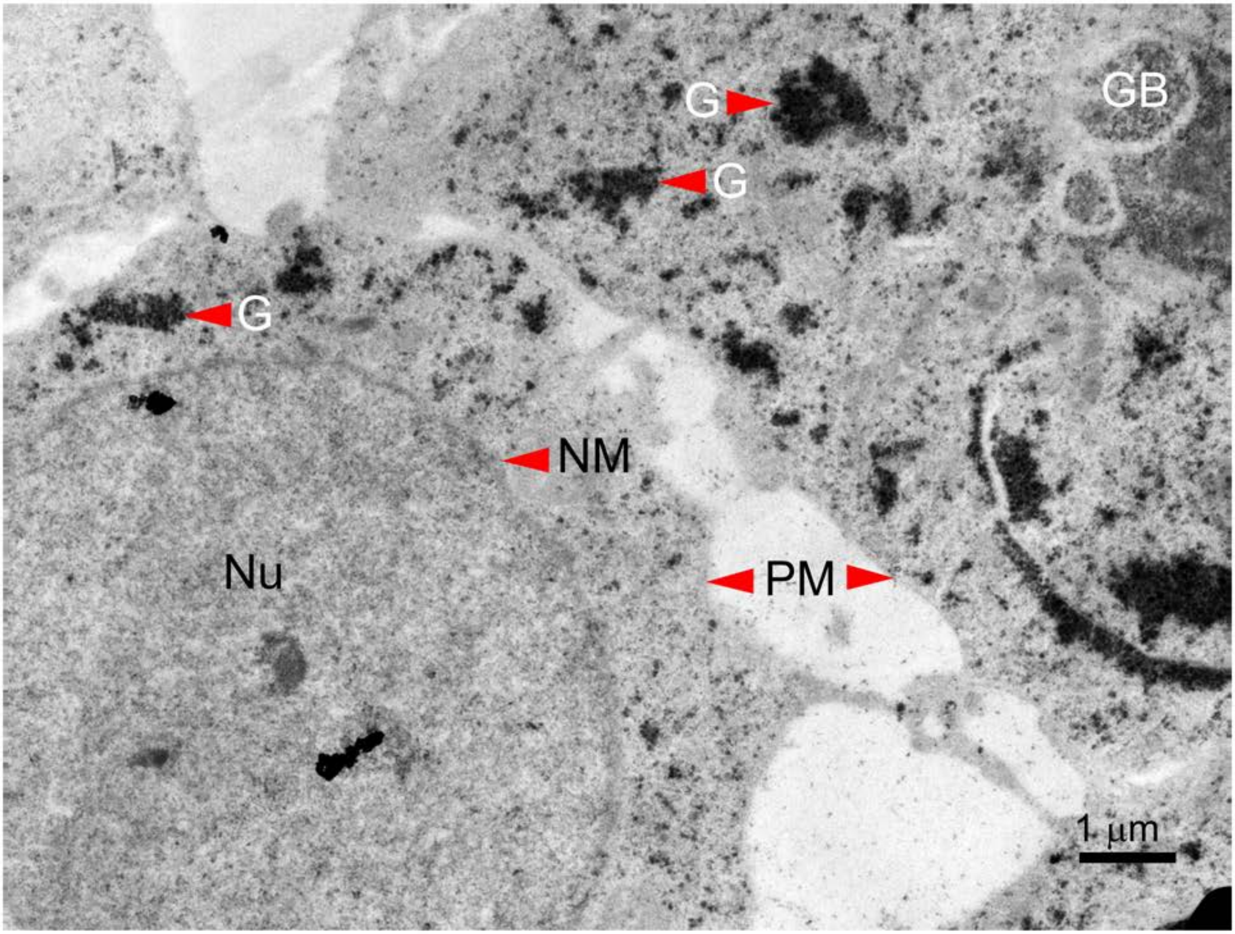

S2 Fig. (H) TEM analysis of glycogen synthesis and the formation of glycogen bodies mediated by GSK-3 inhibition in BC1 iPSCs.
